# Bacteriocin Microcin J25’s antibacterial infection effects and novel non-microbial regulatory mechanisms: differential regulation of dopaminergic receptors

**DOI:** 10.1186/s40104-024-01115-3

**Published:** 2024-11-13

**Authors:** Lijun Shang, Fengjuan Yang, Qingyun Chen, Ziqi Dai, Guangxin Yang, Xiangfang Zeng, Shiyan Qiao, Haitao Yu

**Affiliations:** 1 Department of Animals Sciences, College of Animal Sciences, Jilin University, Changchun, 130062 China; 2 State Key Laboratory of Animal Nutrition and Feeding, Ministry of Agriculture and Rural Affairs Feed Industry Centre, China Agricultural University, Beijing, 100193 P.R. China; 3 Beijing Bio-feed additives Key Laboratory, Beijing, 100193 P.R. China

**Keywords:** Arachidonic acid, Dopaminergic receptors, Enteric nervous system, Macrophage, Microbiota, Microcins

## Abstract

**Background:**

The antibacterial and immunomodulatory activities of bacteriocins make them attractive targets for development as anti-infective drugs. Although the importance of the enteric nervous system (ENS) in the struggle against infections of the intestine has been demonstrated, whether it is involved in bacteriocins anti-infective mechanisms is poorly defined.

**Results:**

Here, we demonstrated that the bacteriocin Microcin J25 (J25) significantly alleviated diarrhea and intestinal inflammation in piglets caused by enterotoxigenic *Escherichia coli* (ETEC) infection. Mechanistically, macrophage levels were significantly downregulated after J25 treatment, and this was replicated in a mouse model. Omics analysis and validation screening revealed that J25 treatment induced significant changes in the dopaminergic neuron pathway, but little change in microbial structure. The alleviation of inflammation may occur by down-regulating dopamine receptor (DR) D1 and the downstream DAG-PKC pathway, thus inhibiting arachidonic acid decomposition, and the inhibition of macrophages may occur through the up-regulation of DRD5 and the downstream cAMP-PKA pathway, thus inhibiting NF-κB.

**Conclusions:**

Our studies’ findings provide insight into the changes and possible roles of the ENS in J25 treatment of ETEC infection, providing a more sophisticated foundational understanding for developing the application potential of J25.

**Supplementary Information:**

The online version contains supplementary material available at 10.1186/s40104-024-01115-3.

## Introduction

Bacteriocins are antibacterial peptides or proteins which are effective weapons of multicellular organism immune defenses, with strong antibacterial, antiviral and antifungal activities [1, 2]. Due to their narrow activity spectrum and unique killing mechanisms, the side effects on the natural healthy microbiota and bacterial resistance evolution are much more diminutive than those of antibiotics [2–4]. In addition, some bacteriocins have evolved functions such as immunoregulation [5]. These properties make bacteriocins attractive as anti-infective drugs in both clinical and veterinary settings.

At present, most screening of bacteriocins relies on the evaluation of the inhibitory activity in vitro by agar-spot assay, agar-well assay or spot-on-lawn assay [6, 7]. However, the anti-infective mechanism of bacteriocins, such as in cellular or animal infection models, has been found to be related to a variety of factors. Firstly, due to its bactericidal properties, many studies have explored the underlying mechanisms from a microbiological perspective. For example, microcin has been found to be effective in treating pathogen-infected mice by limiting the expansion of pathogens, such as adherent-invasive *Escherichia coli* and the related pathogen *Salmonella enterica* [8]. In addition, bacteriocins are drawing attention as potential immune-modulating agents [9]. Nisin has been shown to increase the number of macrophages in mice peripheral blood, and it has been suggested that it could be used as an immunomodulatory agent in the pork industry [10, 11]. Similarly, it has been suggested that bacteriocin-producing strains may function as probiotics against infection via immunomodulation [10–12]. However, we still lack evidence that combines gut microbiota and immunity to explain the prevention mechanisms in the enteric infection of bacteriocins, especially in humans and other mammals.

In addition to the above factors, increasing evidence suggests that the enteric nervous system (ENS) plays an important role in defending against enteric pathogens and inflammation [13–15]. In addition, interactions among ENS-immune-gut microbes have also been gradually discovered [14, 16–18]. Nevertheless, there has been little research on the effects of bacteriocins on the ENS during anti-infection processes. Collectively, a more comprehensive understanding of the anti-infective mechanisms of bacteriocins is needed.

Microcin J25 (J25) is one of the most studied Gram-negative bacteriocins, with strong bactericidal activity primarily against *Salmonella*, *E. coli* O157:H7 and enterotoxigenic *Escherichia coli* (ETEC) [19, 20]. ETEC is the main cause of diarrhea of both young children and travelers in developing countries [21, 22], and the main pathogen causing postweaning diarrhea in pigs, leading to enormous economic losses [23, 24]. Although J25 has been shown to have therapeutic effects on ETEC-infected cell lines and mice [25, 26], no reports about its effectiveness in the treatment of mammalian infections have been published. In addition, as with many other bacteriocins, a comprehensive and in-depth understanding of its mechanism is lacking.

In this study, we used ETEC-challenged piglets as animal models, and J25 as a typical representative of bacterial-derived antimicrobial peptides (AMPs). Based on previous research, we hypothesized that J25 has a beneficial effect on the piglet diarrhea and intestinal injury caused by ETEC infection. We sought to unravel the mechanisms of the beneficial effects by comprehensively describing the changes in immunity, microbiota, and ENS, as well as their potential interconnections after therapeutic administration of J25. These findings will provide a perspective for explicating the new functions and anti-infection mechanisms of bacterial-derived AMPs, which is conducive to promoting the theoretical research, product development and application of AMPs.

## Materials and methods

The experiments were conducted at the Fengning Swine Research Unit of China Agricultural University (Chengde Jiuyun Agricultural and Livestock Co., Ltd., Hebei, China). All experimental procedures and animal care were approved by the China Agricultural University Animal Care and Use Committee (Beijing, China).

### Preparation of Microcin J25

Microcin J25 was generated in our laboratory using a previously described method [20]. Genscript manufactured the codon-optimized gene coding for J25, and then cloned them into pBR322 to generate a novel expression vector pMJ25. The vector was transformed into *E. coli* BL21, and the recombinant bacteria were cultivated for 20 h in a sucrose-complex medium with 100 µg/L ampicillin in a 10-L fermenter at 37 °C. The supernatant and J25 were collected and purified by an AKTA Purifier System (Amersham Biosciences, Piscataway, NJ, USA), and the purity was above 99.5%. J25 was produced as a lyophilized powder and dissolved in PBS for gavage.

### Bacterial strain and culture conditions

The ETEC strain used in this study (ETEC K88, serotype O149:K91, K88ac) was purchased from the China Institute of Veterinary Drug Control (Beijing, China). The ETEC strain was grown under typical conditions either in Luria-Bertani (LB) broth (Beijing AoBoXing Biotechnology Co., Ltd., Beijing, China) or on LB agar plates at 37 °C.

### Piglet selection

To be eligible for the study, the piglets had to meet the following inclusionary criteria: susceptible to ETEC F4 infection; born from the same litter and weaned; no history of intestinal disease from birth; no history of drug treatment; similar body weight (BW); and equal numbers of barrows and gilts.

The ETEC F4-susceptible piglets were selected on the basis of genotype for F4ab/ac susceptibility [27]. DNA was extracted from tails or blood obtained from piglets 3 d or 20 d after birth, using the method described by the protocol supplied by the manufacturer (TIANamp Genomic DNA Kit, Tiangen Biotech (Beijing) Co., Ltd., China). The PCR of the *MUC4* gene was performed using a 3-min initial denaturation at 95 °C, followed by 35 cycles of 98 °C for 10 s, 58 °C for 5 s and 68 °C for 5 s (LightCycler Real-Time PCR System, Roche, Germany). The primers are listed in Additional file 1: Table S2. The PCR product was digested with FastDigest XbaI (Thermo Fisher Scientific) at 37 °C for 5 min. The size of the PCR product was 367 bp. The resistant samples were indigestible by XbaI, whereas the susceptible samples were digested into 151 bp and 216 bp fragments.

### Experimental design for piglets

After piglet selection, all piglets were susceptible to ETEC F4 and housed individually in stainless-steel holding crates (1.4 m × 0.7 m × 0.6 m). Feed and freshwater were available ad libitum with controlled temperature (23 ± 2 °C) and humidity (55%–65%). Diets (Additional file 1: Table S1) were formulated based on the National Research Council’s nutrient recommendations [28].

For ETEC dose determination, 24 weaned piglets (Duroc × Yorkshire × Landrace, 12 barrows and 12 gilts with an average BW of 10.95 ± 0.10 kg) at the age of 28 d were used in the infection model establishment trial. The pigs were randomly assigned to one of four treatments: normal control group (NC), low dose ETEC challenge group (L-ETEC), medium dose ETEC challenge group (M-ETEC), and high dose ETEC challenge group (H-ETEC), received 100 mL PBS, 10^8^ CFU/mL, 5 × 10^8^ CFU/mL, and 10^9^ CFU/mL ETEC, respectively (Additional file 1: Fig. S2A). The doses were determined by integrating the results of previous studies [27, 29, 30]. Body weight and feed intake were recorded at d 0 and d 5, and blood and fecal samples were taken on d 0, 24 h after ETEC challenge and d 5.

**Fig. 1:**
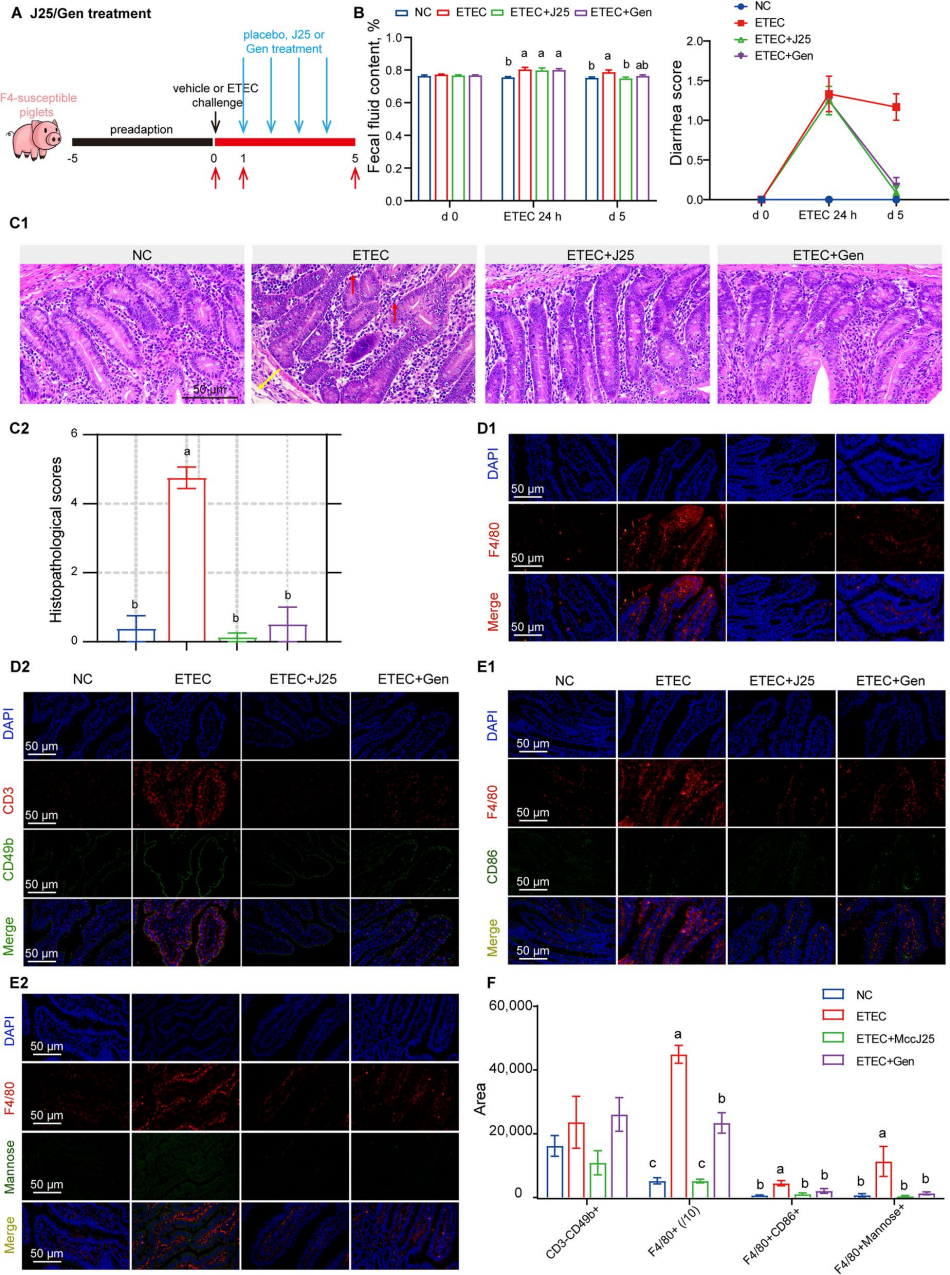
The effect of J25 on clinical symptoms, jejunum morphology and immune cells. **A** Schematic of the experimental design. Red arrows indicate the days on which the samples were collected for analysis. **B** Fecal fluid content analysis (left) and diarrhea score (right). Scoring standards: 0, normal; 1, loose stool; 2, moderate diarrhea; 3, severe diarrhea. Data are presented as mean ± SEM, *n* = 12. **C** Representative images of the jejunum by H&E staining (200×, C1) and histopathological scores (C2) in J25 treated ETEC-challenged piglet experiment. The red arrow indicates inflammatory cell infiltration in the mucosal layer, and the yellow arrow indicates edema status in the submucosa. Data are presented as mean ± SEM, *n* = 8. **D** Representative images of NK and macrophage relative proteins immunoreactivity in the jejunum (D1: F4/80, red; DAPI, blue and D2: CD3, red; α2β3, green; DAPI, blue). **E** Representative images of M1 and M2 subtype macrophage relative proteins immunoreactivity in the jejunum in J25 treated ETEC-challenged piglet experiment. (E1: F4/80, red; CD86, green; DAPI, blue and E2: F4/80, red; Mannose, green; DAPI, blue). **F** Relative immunolabeling quantification of D and E. Data are presented as mean ± SEM, *n* = 8. ANOVA followed by Tukey’s multiple comparisons test; different lowercase letters within each group indicate significantly different values (*P* < 0.05)

Forty-eight piglets as described above with an average BW of 10.87 ± 0.10 kg were used in the therapeutic trial. The piglets were randomly assigned to one of four treatments: (1) normal control group (NC): vehicle challenge, followed by placebo treatment; (2) ETEC group: ETEC challenge, followed by placebo treatment; (3) ETEC + J25 group: ETEC challenge, followed by 1 mg/kg J25 treatment; and (4) ETEC + Gen group: ETEC challenge, followed by 2 mg/kg gentamicin (Gen) treatment (Fig. 1A). The ETEC dose was determined by a pilot experiment. Body weight and feed intake were recorded at d 0 and 5, and blood and fecal samples were taken on d 0, and after the ETEC challenge for 24 h and d 5. After sampling on d 5, pigs (8/group) were humanely euthanized by exsanguination after electrical stunning to obtain samples.

### Experimental design for mice

Female BALB/c mice (6–7 weeks) and γ-ray-sterilized normal feed were obtained from SPF (Beijing) Biotechnology Co., Ltd. The animals were maintained on a 12:12 h light-dark cycle under specific pathogen-free conditions. All animals had ad libitum access to water and feed throughout the experiment. Experiments on animals were performed in accordance with the Animal Care and Use Committee of China Agricultural University.

#### The effects of J25 on ETEC-challenged mice

The procedure was based on our previous study [26]. Infection procedure: the mice were treated with streptomycin (100 µL of a 500 mg/mL solution in sterile water; Sigma-Aldrich, St. Louis, MO, USA) in drinking water 1 d prior to ETEC infection to eradicate any normal resident bacterial flora in the intestinal tract. The streptomycin-treated water also contained fructose (6.7%) (Karo Syrup; ACH Food Companies, Inc, Memphis, TN, USA) to encourage water consumption. The following day, mice were orally inoculated with 5 × 10^10^ colony forming units (CFU) of ETEC. Experimental design: 72 mice (after 3 days of adaption) were randomly divided into 4 groups as follows: (1) normal control (NC), (2) infected control (ETEC), (3) ETEC + J25, and (4) ETEC + gentamicin (Gen). At d 1 and 3 after ETEC infection, the mice were either mock-treated or treated with 9.1 mg/kg of body weight (BW) J25 or 10 mg/kg of BW gentamicin in the same manner. At d 5 post-infection, the mice were euthanized (Additional file 1: Fig. S4A).

#### Peripheral dopaminergic neuron deficit mouse model

6-Hydroxydopamine (6-OHDA, Sigma Aldrich, H116) was dissolved in 0.02% ascorbic acid (Aladdin, Shanghai, China) to prevent oxidation. Then, after 3 d of acclimation, 60 mice were randomly divided into 5 groups and administered the solution intraperitoneally, 0, 4, 8, 10, and 12 µg 6-OHDA per mouse. Six mice from each group were euthanized on d 1 and 6 post-infection.

#### Functional validation of dopaminergic neurons

First, an enteric 6-OHDA-lesion model was established (Additional file 1: Fig. S8). Twenty-four mice were randomly assigned to 1 of 3 treatments: (1) normal control group (NC): vehicle intraperitoneal injection and challenge, followed by placebo treatment; (2) DA^− ^+ ETEC group: 6-OHDA intraperitoneal injection and an ETEC challenge, followed by a placebo treatment; and (3) DA^− ^+ ETEC + J25 group: 6-OHDA intraperitoneal injection and an ETEC challenge, followed by a 9.1 mg/kg J25 treatment.

#### Functional validation of arachidonic acid

Forty mice were randomly assigned to one of five treatment groups: (1) normal control group (NC): vehicle challenge, followed by placebo treatment; (2) ETEC group: ETEC challenge, followed by placebo treatment; (3) and (4) ETEC + low or high dose Zileuton group (LZ or HZ group): ETEC challenge, followed by 50 mg/kg or 100 mg/kg Zileuton treatment; and (5) ETEC + J25 group (J25): ETEC challenge, followed by 9.1 mg/kg J25 treatment. The doses of the ETEC and J25 were determined by our previous study [26].

### ELISA

We centrifuged the blood to obtain serum and homogenized the jejunum with normal saline to obtain the supernatant. The levels of tryptophan hydroxylase (TPH), IL-1β, IL-8, IL-10, TNF-α, DAO and D-lactate were determined using ELISA kits (Nanjing Jiancheng Bioengineering Institute, Nanjing, China). The kit assays were carried out according to the protocol supplied by the manufacturer. The absorbance was read using a multimode microplate reader (iMark, Bio-Rad, USA).

### Fecal indexes

The fecal fluid content was determined by desiccation in an oven (50 °C, 6 h), and weighing before and after desiccation [31]. Fecal score: 0, normal; 1, loose stool; 2, moderate diarrhea; 3, severe diarrhea [32]. Quantification of fecal microflora was performed by qRT‒PCR, and the primers are listed in Additional file 1: Table S2.

### Ultraviolet colorimetry

Glutaminase (GLS) and choline acetyltransferase (ChAT) content in jejunal tissue was determined by ultraviolet colorimetry according to the instructions provided by the supplier (Nanjing Jiancheng Bioengineering Institute, Nanjing, China).

### Histology and immunofluorescence

Histological damage was quantitatively assessed as described in Additional file 1: Table S3. The sum of the four subscores results in a combined score ranging from 0 (no changes) to 12 (widespread cellular infiltrates and extensive tissue damage). Each section in each group was selected and photographed at 40 × field of view. Five complete villi were selected from each section, and the villus length (µm) and crypt depth (µm) were measured.

The immunofluorescence staining sections were observed under a Nikon Eclipse C1 microscope, and the images were captured using a Nikon DS-U3. Three fields were randomly selected from each section and photographed at 100 × view. We measured the positive areas with Image-Pro Plus 6.0 (Media Cybernetics, Inc., Rockville, MD, USA). Enteric glial cells (EGCs) were quantified using volume densities (Vv) [33–35], which were defined as the quotient of the volume of interest divided by the reference volume, irrespective of the staining intensity ( Additional file 1: Table S4).

### Microbiota composition by 16S rRNA sequencing analysis

We obtained jejunal mucosal samples for sequencing. Genomic DNA was extracted using the TGuide S96 Magnetic Soil/Stool DNA Kit (Tiangen Biotech (Beijing) Co., Ltd., China). The V3–V4 regions of the bacterial 16S rRNA gene were amplified with universal primers 338F (5′-ACTCCTACGGGAGGCAGCAG-3′) and 806R (5′-GGACTACHVGGGTWTCTAAT-3′). PCR amplicons were purified with Agencourt AMPure XP Beads (Beckman Coulter, Indianapolis, IN, USA) and quantified using the Qubit dsDNA HS Assay Kit and Qubit 4.0 Fluorometer (Invitrogen, Thermo Fisher Scientific, Oregon, USA). Purified amplicons were pooled in equimolar amounts and sequenced on an Illumina NovaSeq 6000 (Illumina, Santiago, CA, USA). Raw data were primarily filtered by Trimmomatic (version 0.33) [36]. The identification and removal of primer sequences was processed by Cutadapt (version 1.9.1) [37]. Any chimeras were removed with USEARCH (version 10) assembly and UCHIME (version 8.1) [38, 39]. Operational taxonomic units (OTUs) were clustered with 97% similarity by USEARCH (version 10.0) [36]. The OTUs’ taxonomy annotation was performed based on the Naive Bayes classifier in QIIME2 using the SILVA database (release 132) with a confidence threshold of 70% [38, 40].

### LC‒MS/MS analysis

The jejunum tissues were collected for metabolomics analysis. Metabolites with variable importance in projection (VIP) of ≥ 1 and *P* < 0.05 were regarded as statistically significant (differentially expressed metabolites). Pathway analysis was performed by integrating a hypergeometric test and topology analysis.

The LC–MS/MS system for metabolomics analysis was composed of a Waters Acquity I-Class PLUS ultrahigh-performance liquid chromatogram with Waters Xevo G2-XS QTof high-resolution mass spectrometer. Positive ion mode: mobile phase A: 0.1% formic acid aqueous solution; mobile phase B: 0.1% formic acid acetonitrile. Negative ion mode: mobile phase A: 0.1% formic acid aqueous solution; mobile phase B: 0.1% formic acid acetonitrile. Injection volume: 1 µL. The Waters Xevo G2-XS QTof high-resolution mass spectrometer can collect primary and secondary mass spectrometry data in MSe mode under the control of the acquisition software (MassLynx V4.2, Waters).

The raw data collected using MassLynx V4.2 were processed by Progenesis QI for peak extraction, peak alignment and other data processing operations, based on the Progenesis QI online METLIN database and Biomark’s self-built library for identification. At the same time, theoretical fragment identification and mass deviation were all within 100 ppm. The original peak area information was normalized to the total peak area, and we used principal component analysis and Spearman correlation analysis to judge the samples’ repeatability within the group and the quality control samples.

### Western blot

Jejunum tissue proteins were extracted using RIPA lysis Buffer (Bosterbio, USA) and protease inhibitor cocktail (Apexbio, China). Proteins were quantified using a BCA protein assay kit (Beyotime, Shanghai, China). Approximately 30 µg of protein was separated using 10% SDS‒PAGE and then transferred to PVDF membranes. The membranes were incubated with primary Abs (ALDH1A1, GAT1, PKA, PKC, p65, p-p65, Cytp450, Lox and Cox, Additional file 1: Table S5) overnight at 4 °C after blocking with 5% skim milk powder or BSA and then incubation with secondary Abs (Table S5) for 1 h at room temperature. After 3 washes in TBST, ECL Plus™ solution (Millipore, USA) was added, and images were taken in an imaging system (ChemiDoc™ XRSplus, Bio-Rad) using GAPDH as the internal control.

### Statistical analysis

We conducted statistical analysis in Prism (GraphPad 9.0.1). Data were first checked for a normal distribution, and then plotted in the figures as the mean ± SEM. For any experiments containing more than two relative groups, one-way ANOVA followed by Dunnett’s or Tukey’s multiple comparisons post hoc test was performed. Differences in bacterial data were evaluated by the Wilcoxon rank‒sum test or Kruskal‒Wallis H test. PICRUSt software was used to compare the species composition information obtained from the 16S sequencing data to infer the functional gene composition. Random forest analysis was performed using R v3.1.1 (random forest v4.6-10) to obtain any key species that had important effects on intergroup differences. Python 2.7.8 (scipy-0.14.1) and Spearman were used to calculate the correlation coefficient between species and phenotypes, which were visualized in the form of a heatmap. *P* values < 0.05 were considered statistically significant.

## Results

### J25’s effects on ETEC infection in piglets

Prior to the two trials, 24 and 48 piglets with F4 susceptibility were selected from a total of 115 and 147 piglets, respectively. In each group, half of the piglets were male and the other half were female (Fig. S1). Following the challenge, both the medium dose ETEC (M-ETEC) and high dose ETEC (H-ETEC) groups showed high fecal fluid content, diarrhea scores and decreased growth performance (Fig. S2 and Table 1). Based on important infection-associated parameters, we chose a 100 mL, 5 × 10^8^ CFU/mL dose to build a model with moderate symptoms. Diarrhea and inflammation in this model persisted for at least 5 d, which constituted the entire trial period.

**Table 1:** Effect of ETEC infection on the growth performance of weanling pigs

Item	NC	L-ETEC	M-ETEC	H-ETEC	SEM	*P*-value
Initial BW, kg	10.93	10.93	10.96	10.96	0.59	> 0.99
D 0 BW (after acclimatization), kg	12.03	12.57	12.17	12.28	0.67	0.96
D 5 BW (end of the trial), kg	14.70	14.13	13.77	13.50	0.68	0.66
Weight gain, kg	2.67^a^	1.57^ab^	1.60^ab^	1.23^b^	0.28	0.01
ADG, g	533^a^	313^ab^	320^ab^	247^b^	55	0.01
ADFI, g	738	728	675	674	25	0.21
G:F	0.73^a^	0.43^ab^	0.49^ab^	0.37^b^	0.08	0.05

*BW* Body weight, *ADG* Average daily gain, *ADFI* Average daily feed intake, *G:F* Feed:gain, *ETEC* Enterotoxigenic *Escherichia coli*, *NC* Normal control group, *L-ETEC *Low dose ETEC challenge group, *M-ETEC* Medium dose ETEC challenge group, *H-ETEC* High dose ETEC challenge group

^a,b^Means in the same row with different superscripts differ significantly (*P* < 0.05)

ETEC infection promotes small intestinal lesions and alters intestinal permeability, disrupting absorption and secretion. This results in diarrhea and decreased growth performance [41–44]. Given that ETEC infection causes the above characteristics, we assessed growth performance, diarrhea, and jejunal pathology, etc., after both J25 and gentamicin (Gen) treatment.

In both the J25 and Gen treatment groups, infected piglets showed some improvement in growth performance (Table 2). The improvement was also observed in fecal score, but only J25 treatment significantly reduced fecal fluid content (Fig. 1B). Histological analysis showed obvious impairment in the jejunum of ETEC-challenged piglets, as reflected by severely damaged mucosal structures and enormous inflammatory cell infiltration (Fig. 1C). In contrast, both the ETEC + J25 and ETEC + Gen groups showed an intact mucosal layer, and clear and normal tissue structure (Fig. 1C); at the same time, a decreased crypt depth and increased ratio of villus height to crypt depth (VCR) were also observed in the ETEC + J25 group (Fig. S3A), confirming the repair effect of J25 on intestinal infection. Serum enzyme activity measurements of DAO and D-lactate confirmed that J25 exhibited decreased intestinal permeability function (Fig. S3B). In terms of circulating cytokines in serum, a reduction in the IL-1β and TNF-α levels was observed in the ETEC + J25 group, compared to the ETEC group (Fig. S3C); in terms of cytokines in the jejunal tissue, a reduction in the TNF-α level was observed in the ETEC + J25 group, compared to the ETEC group (Fig. S3D). Collectively, we provided preliminary evidence for the anti-ETEC infective effect of J25 in mammals (piglets).

**Table 2:** Effects of Microcin J25 and gentamicin on ETEC infected weanling pigs’ growth performance

Item	NC	ETEC	ETEC + J25	ETEC + Gen	SEM	*P*-value
Initial BW, kg	10.85	10.93	10.83	10.86	0.25	0.99
D 0 BW (after acclimatization) kg	11.92	11.74	11.96	11.89	0.25	0.82
D 5 BW (before euthanasia) kg	14.58^a^	13.42^b^	14.07^ab^	13.93^ab^	0.28	0.03
Weight gain, kg	2.67^a^	1.67^b^	2.11^b^	2.04^b^	0.10	< 0.01
ADG, g	532^a^	334^b^	423^ab^	406^b^	23	< 0.01
ADFI, g	737	667	692	726	22	0.06
G:F	0.73^a^	0.51^b^	0.61^ab^	0.57^b^	0.03	< 0.01

*BW* Body weight, *ADG* Average daily gain, *ADFI* Average daily feed intake, *G:F* Feed:gain, *ETEC* Enterotoxigenic *Escherichia coli*, *NC* Normal control group, *L-ETEC* Low dose ETEC challenge group, *M-ETEC* Medium dose ETEC challenge group, *H-ETEC* High dose ETEC challenge group

^a,b^Means in the same row with different superscripts differ significantly (*P* < 0.05)

In addition, ETEC challenge significantly increased fecal *E. coli* populations, but had little effect on the total bacteria number (Fig. S3E). Compared with the ETEC group, J25 significantly reduced fecal *E. coli* populations, but neither J25 nor Gen had any effect on the total bacteria number (Fig. S3E).

### J25 treatment restores ETEC-challenged piglets’ macrophage status

ETEC infection results in mild intestinal inflammation, increased innate immune gene expression and macrophages infiltration in the gut mucosa [21, 45–47]. To examine whether intestinal innate immunity is involved in the anti-ETEC infection effect of J25, we detected jejunal natural killer cells (NK cells, CD3^−^CD49b^+^) and macrophages (F4/80^+^) with an immunofluorescence assay. A significant increase in macrophages but no significant change in NK cells in ETEC-challenged piglets was observed (Fig. 1D and F). J25 treatment inhibited the expression of macrophage markers to the NC level, whereas the inhibitory effect of Gen treatment was weaker (Fig. 1F). Given that flow cytometry is more accurate in detecting intestinal innate immune cells, we repeated the experiment using a mouse model to assess trends in NK cells and macrophages (Fig. S4). We were pleasantly surprised to observe the same trend (Fig. S4F and G). Collectively, these findings indicate that J25 affects intestinal innate immunity by inhibiting macrophages, and these findings are reproducible in different species.

Macrophages are mainly divided into M1 and M2 macrophage subtypes. We assessed changes in subtyping by detecting CD86 (M1 marker) and mannose (M2 marker) expression. In the ETEC group, we observed significant increases in both CD86 and mannose expression (Fig. 1E and F). Notably, after J25/Gen treatment, the expression of CD86 and mannose were downregulated (Fig. 1E and F). Thus, J25 effectively downregulated the ETEC-induced macrophage infiltration to normal levels.

### J25 caused little change in microbial structure

Because of the close contact between the innate immune system and the gut microbiota [48, 49], and because J25 alleviates dextran sulfate sodium salt (DSS)-induced colitis by modifying the gut microbiota [50], we sought to identify whether J25/Gen treatment causes microbiota changes in ETEC-challenged piglets.

**Fig. 2:**
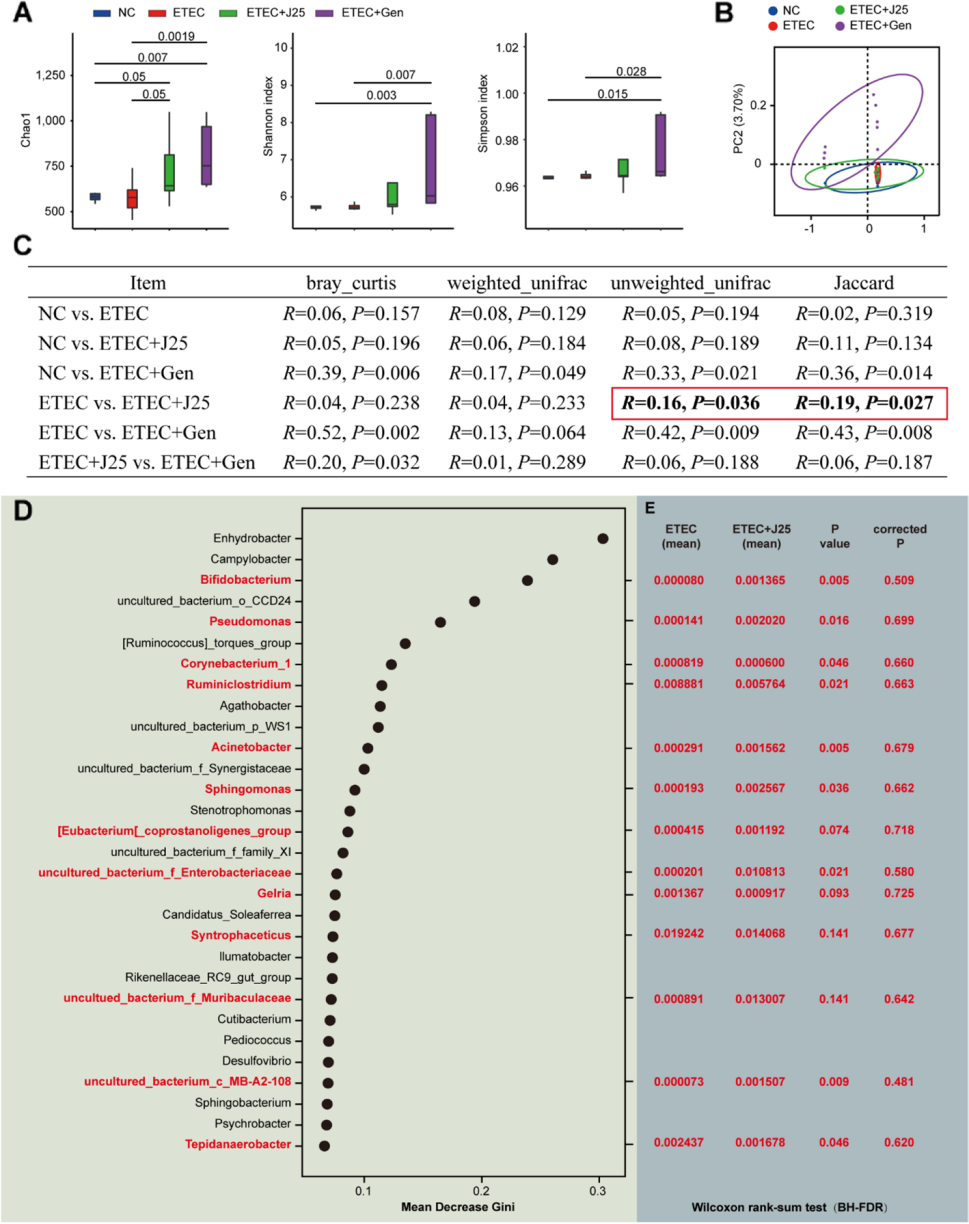
J25 caused little change in the overall microbiota composition. **A** Alpha diversity comparisons of the microbial communities. Data are presented as mean ± SEM, *n* = 8. The numbers on the horizontal lines indicate the *P*-values using a paired Student’s *t*-test. **B** PCoA of 16S genes. Using an OTU definition of 97% similarity, and based on Bray_Curtis. **C** Distance comparisons between groups. NMDS followed by ANOISM was used for each pairwise comparison. **D** The most important biomarkers identified by random-forest classification in the ETEC and the ETEC + J25 groups, with the biomarker taxa ranking in descending order of importance in terms of model accuracy. Genera that coincided with the association analysis results (Fig. S5) are marked in red. **E** Relative abundance (%) of marker bacteria, Wilcoxon rank-sum test, BH-FDR corrected

The piglet microbiota was assessed by sampling jejunal mucosa from four groups. Through 16S RNA sequencing, we found that only the ETEC + Gen group had increases on the Shannon and Simpson indexes, reflecting both species richness and evenness, compared with the ETEC group; both the ETEC + J25 group and the ETEC + Gen group had increases on the species richness index Chao1 (Fig. 2A), which may have been because Chao1 is more sensitive to rare species. Our results indicated that Gen increased species richness but decreased community evenness. J25 increased some rare species, but did not affect community evenness.

To estimate the relatedness of the microbial communities, we calculated distances between samples by using bray_curitis (Fig. 2B). Principal coordinate analysis (PCoA) revealed clear clustering between the ETEC + Gen group and the other 3 groups, but presented no difference in microbial communities among the NC, ETEC, and ETEC + J25 groups (Fig. 2B). To rule-out the possibility that the lack of difference between the 3 groups had been caused by the large difference between the ETEC + Gen group and these 3 groups, we performed ANOISM analyses (95% confidence, Fig. 2C). Under unweighted calculation conditions (Jaccard and unweighted_unifrac), we found a significant difference in community composition between the ETEC and J25 groups (Fig. 2C). These data suggest that the difference between the ETEC and ETEC + J25 groups was that J25 increased some new species, but these species were all of low abundance.

To measure functional differences between the gut microbiota of the ETEC group and that of the ETEC + J25 group, we predicted metabolic potential using PICRUSt2. The results showed little difference in functional composition between the ETEC group and the ETEC + J25 group (Fig. S5). This is not surprising, as most community differences between the two groups lie in a handful of low-abundance species.

To clarify the functions of these low-abundance differential species, we related the phenotypes to the microorganisms while accounting for potential correlated factors such as microorganism importance measures or microorganism difference tests. Environmental factor screening was performed based on variance inflation factor (VIF) analysis (threshold < 10). All four important parameters (fecal fluid content, macrophages, and M1 and M2 subtypes) met the requirements (Table S6). Then, correlations between four important parameters and the microbiota were analyzed. A total of 44 genera were significantly associated with the parameters (Fig. S6). Of these, 13 genera were identified as biomarkers by random forest analysis (Fig. 2D). We then tested whether any individual genus from the 13 genera was significantly different between the two groups. To identify any taxa differing between the ETEC group and the ETEC + J25 group, we used the Wilcoxon rank-sum test. To our surprise, we found no differences in any genus abundance with a *P* < 0.05 after BH-FDR correction (Fig. 2E). Taken together, these results suggest that although richness increases after J25 treatment, changes in specific gut bacterial species are rare.

### Enteric nervous system changes following ETEC challenge or J25/Gen treatment

There is increasing evidence that the crosstalk between the enteric nervous system (ENS) and innate immune system regulates host homeostasis and immunity, and resists pathogenic microbial invasion [15, 16]. Given that ETEC challenges altered mucosal integrity and macrophage responses in the jejunum, we further tested how ENS responses changed as a result of ETEC challenge or J25/Gen treatment.

**Fig. 3:**
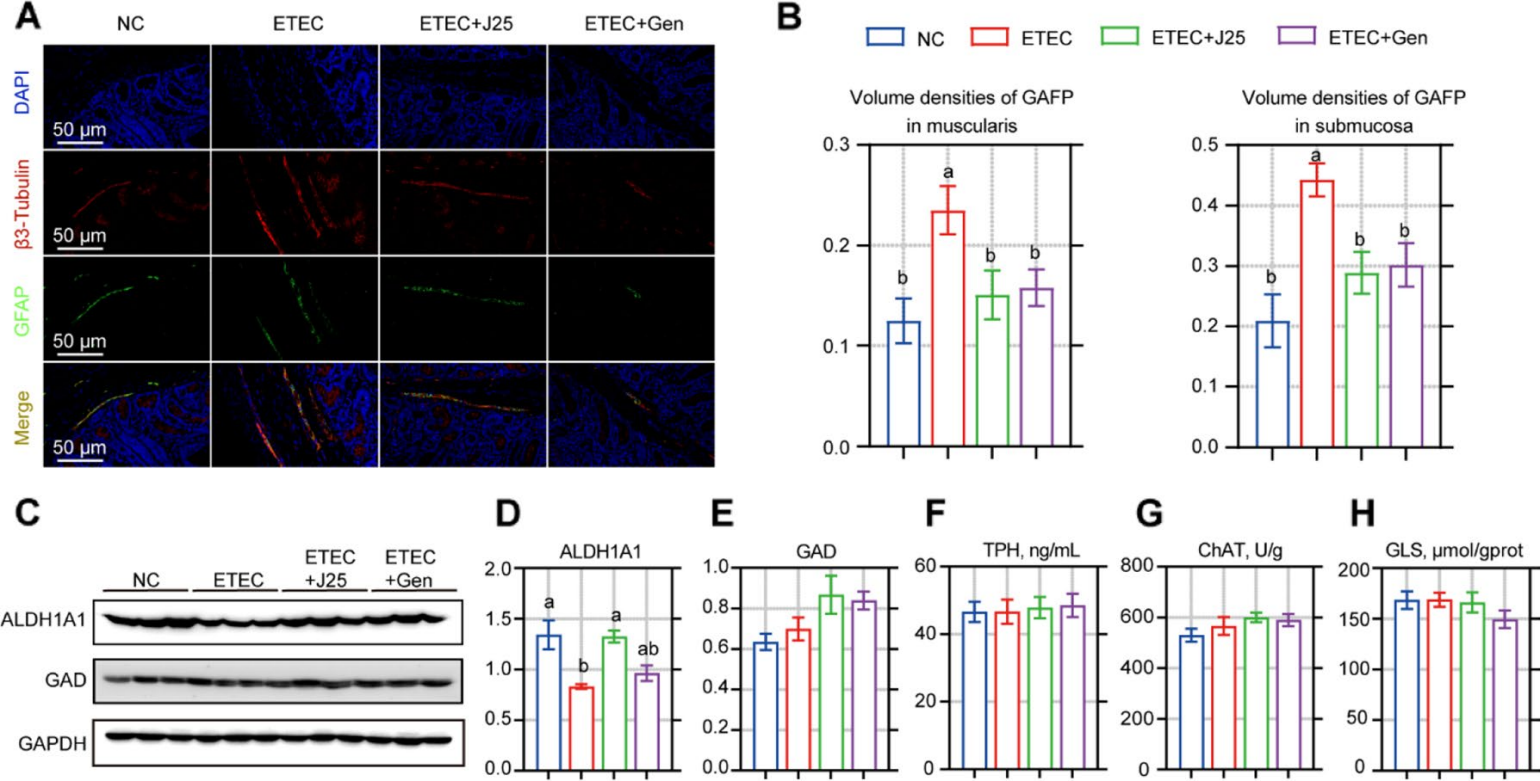
J25 treatment altered the enteric nervous system responses in ETEC-challenged piglets. **A** Representative images of β3-tubulin (red), GAFP (green), and DAPI (blue). **B** Immunolabeling quantification. Measurements were performed using volume density (Vv), which is the quotient from the volume of interest divided by the reference volume, as described in Table S5. *n* = 8. **C–H** Enteric neuron marker detection. ALDH1A1: acetaldehyde dehydrogenase 1A1 (marker for dopaminergic neurons); GAD: glutamic acid decarboxylase (marker for GABAergic neurons); TPH: tryptophan hydroxylase (marker for serotonergic neurons); ChAT: choline acetyltransferase (marker for cholinergic neurons); Gls: glutaminase (marker for glutamatergic neurons). *n* = 3 in C–D and *n* = 8 in F–H. Data are presented as mean ± SEM. ANOVA followed by Tukey’s multiple comparisons test; different lowercase letters within each group indicate significantly different values (*P* < 0.05)

ETEC challenge caused an increase in enteric glial cells (EGCs) in both the submucosa and the muscularis; following J25 treatment, we found a statistically significant decrease in EGCs (Fig. 3A and B). There are five types of mature enteric neurons: glutamatergic neurons, GABAergic neurons, dopaminergic neurons, serotonergic neurons, and cholinergic neurons. Metabolomic results showed that J25 treatment had significant effects on all five neuronal pathways, compared to the ETEC group (Fig. S7). To further verify this result, we examined the markers of these neuronal pathways (Fig. 3C–H). Only a dopaminergic neuron marker (ALDH1A1) was found to be significantly elevated (Fig. 3C and D). This may have been because in the metabolomic results from the serotonergic, cholinergic and GABAergic neural pathways, the transmitters did not change; in the glutamatergic neural pathway, changes in substances other than transmitters coincided with the dopamine (DA) pathway (Fig. S7). Taken together, these findings suggest that ETEC challenge increased the EGCs in the submucosa, but decreased the dopaminergic neuron-related pathways, whereas J25 treatment reversed these changes.

### J25 downregulates macrophages and EGCs via the DA-PKA-NF-κB axis

**Fig. 4:**
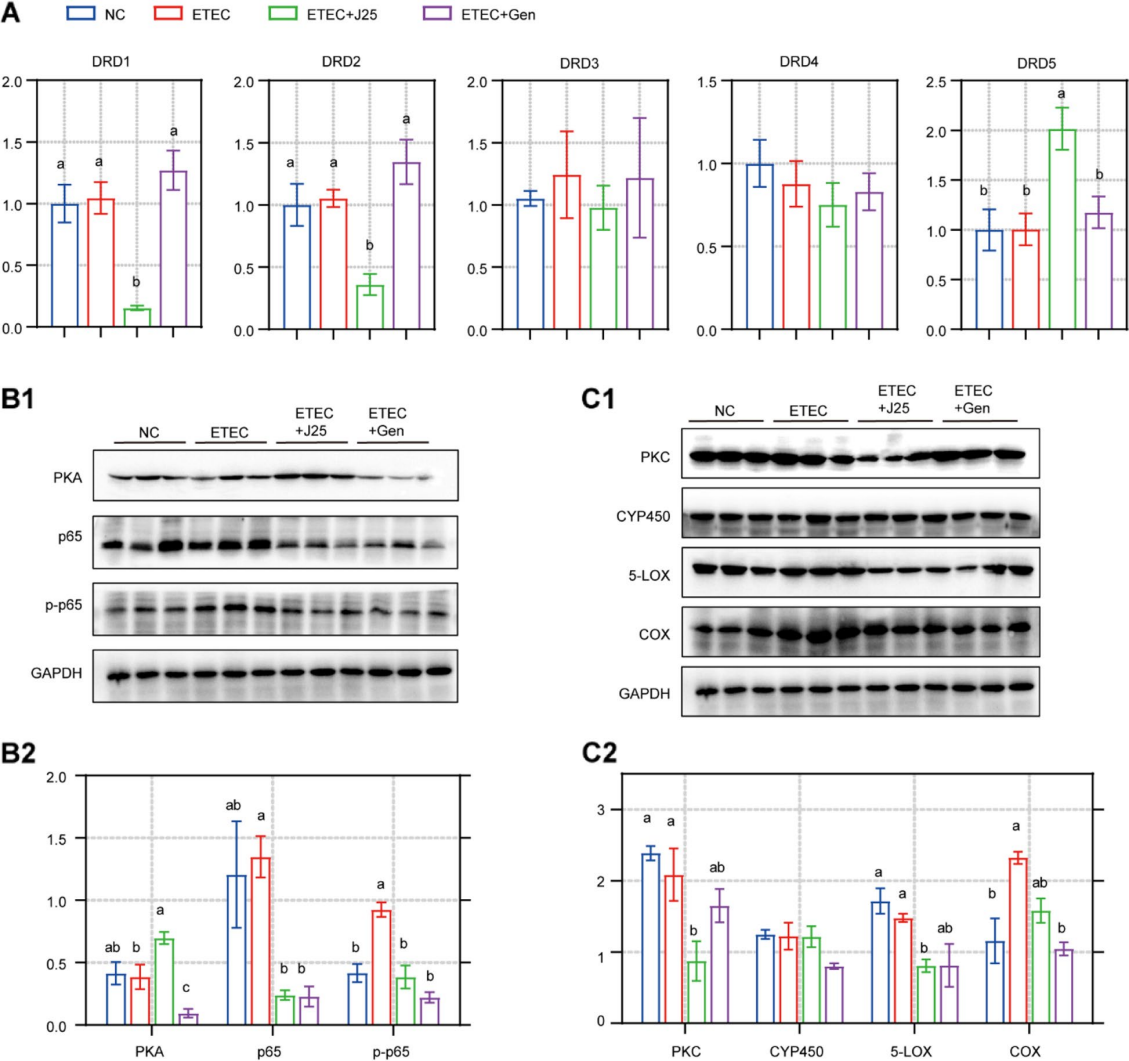
J25 regulates macrophages and inflammation through dopamine and dopamine receptor downstream pathways. **A** Dopamine receptor mRNA expression levels. Data are presented as mean ± SEM, *n* = 8. ANOVA followed by Tukey’s multiple comparisons test; different lowercase letters within each group indicate significantly different values (*P* < 0.05). **B** Protein levels and data statistics in DRD5 downstream pathways. **C** Protein levels and data statistics in DRD1 downstream pathways. Data are presented as mean ± SEM, *n* = 3. ANOVA followed by Tukey’s multiple comparisons test; different lowercase letters within each group indicate significantly different values (*P* < 0.05)

Dopamine receptors (DRs) are divided into D1-class (DRD1 and 5) and D2-class (DRD2, 3 and 4) by their positive and negative regulation of cAMP-PKA [51–55]. Since cAMP was significantly upregulated in the ETEC + J25 group (Fig. S7D), it was reasonable to speculate that the DRs had been altered. To test our hypothesis, we analyzed DRD1–5 gene expression in the jejunum. The results indicated significantly higher mRNA expression of *DRD5* and lower mRNA expression of *DRD2* in the ETEC + J25 group than in the ETEC group (Fig. 4A). However, the mRNA expression of *DRD1* decreased significantly (Fig. 4A). Next, we explored whether elevated D5 in the D1-class alone could cause the upregulation of cAMP-PKA. Consistent with our speculation, we observed significantly upregulated PKA in the ETEC + J25 group (Fig. 4B). Collectively, J25 upregulated cAMP-PKA via DA-DRD5.

cAMP-PKA is critical for inhibiting NF-κB activation [51, 56, 57], while NF-κB activation plays a crucial role in macrophage and EGC infiltration [58–64]. In view of the effect of J25 on macrophages and EGCs, we investigated the changes in NF-κB. We found that J25 markedly increased the p65 and p-p65, both of which are activation indexes of NF-κB (Fig. 4B). Taken together, these data suggest that J25 inhibits cAMP-PKA-mediated NF-kB signaling and subsequent macrophages and EGCs through DA-DRD5.

### J25 downregulates arachidonic acid metabolism via the DA-DAG-PKC axis

Next, we explored the effects of DRD1 reduction. The metabolomic results showed that DAG, which is downstream of DRD1, had significantly decreased in the ETEC + J25 group, compared to the ETEC group (Fig. S7C). Similar to the cAMP-PKA pathway, PKC is the major target of DAG [65, 66]. PKC also plays an important role in the regulation of PLA2, a key enzyme that promotes arachidonic acid (AA) metabolism [67, 68]. Additionally, J25 treatment in ETEC-challenged piglets downregulated AA metabolism, resulting in lower levels of leukotriene C4 and HpETE (Fig. S7E and F). Thus, we hypothesized that PKC would be altered and play an important role in the above processes. To test our hypothesis, we analyzed PKC and 3 key enzymes for AA metabolism (CYP450, 5-LOX, and COX) [69, 70]. Compared with the ETEC group, J25 treatment reduced the PKC and 5-LOX levels, but affected neither the CYP450 nor the COX levels (Fig. 4C). These findings imply that J25‒induced downregulation of DRD1 inhibits downstream DAG-PKC, thereby inhibiting AA metabolism via 5-LOX.

### J25 first acts on dopaminergic neurons

**Fig. 5:**
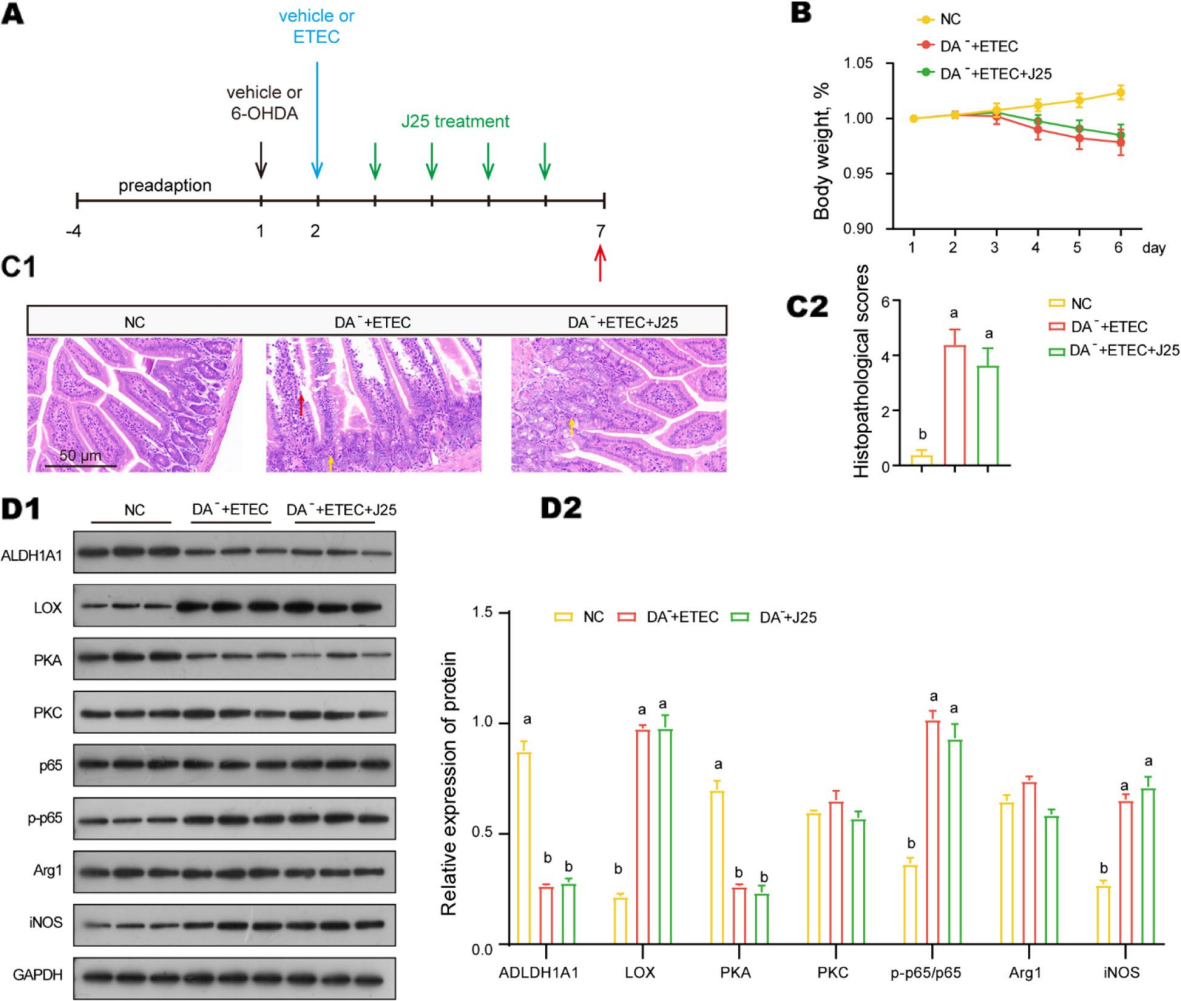
Dopaminergic neurons are critical in the anti-infective mechanism of J25. **A** Schematic of the experimental design. Red arrows indicate the days on which the samples were collected for analysis. **B** Body weight changes (relative to original weight, set as 100%). Data are presented as mean ± SEM, *n* = 8. **C** Representative images of the jejunum by H&E staining (200×) and histopathological scores. Data are presented as mean ± SEM, *n* = 8. **D** Protein levels and data statistics in dopaminergic neuron downstream pathways. Data are presented as mean ± SEM, *n* =3. ANOVA followed by Tukey’s multiple comparisons test; different lowercase letters within each group indicate significantly different values (*P* < 0.05)

To explore dopaminergic neurons’ important role in the anti-infection mechanism of J25, we infected intestinal dopaminergic neuron degeneration mice with ETEC, then with or without J25 treatment (Fig. 5A). Intestinal dopaminergic neuron degeneration did not affect body weight or intestinal tissue health in mice (Fig. S8A and C); however, it mitigated J25’s ability to alleviate ETEC-induced body weight loss and intestinal tissue injury (Fig. 5B and C). The trends of the PKA-NF-κB and PKC-LOX pathways, which as mentioned were regulated by J25 in normal mice, were not present in the dopaminergic neuron degeneration mice (Fig. 5D). Similarly, the downregulation of ETEC-induced macrophage infiltration and AA-LOX activation was diminished (Fig. 5D). These findings imply that J25 acts first on dopaminergic neurons.

### Impaired arachidonic acid metabolism inhibits the development of macrophage inflammation

**Fig. 6:**
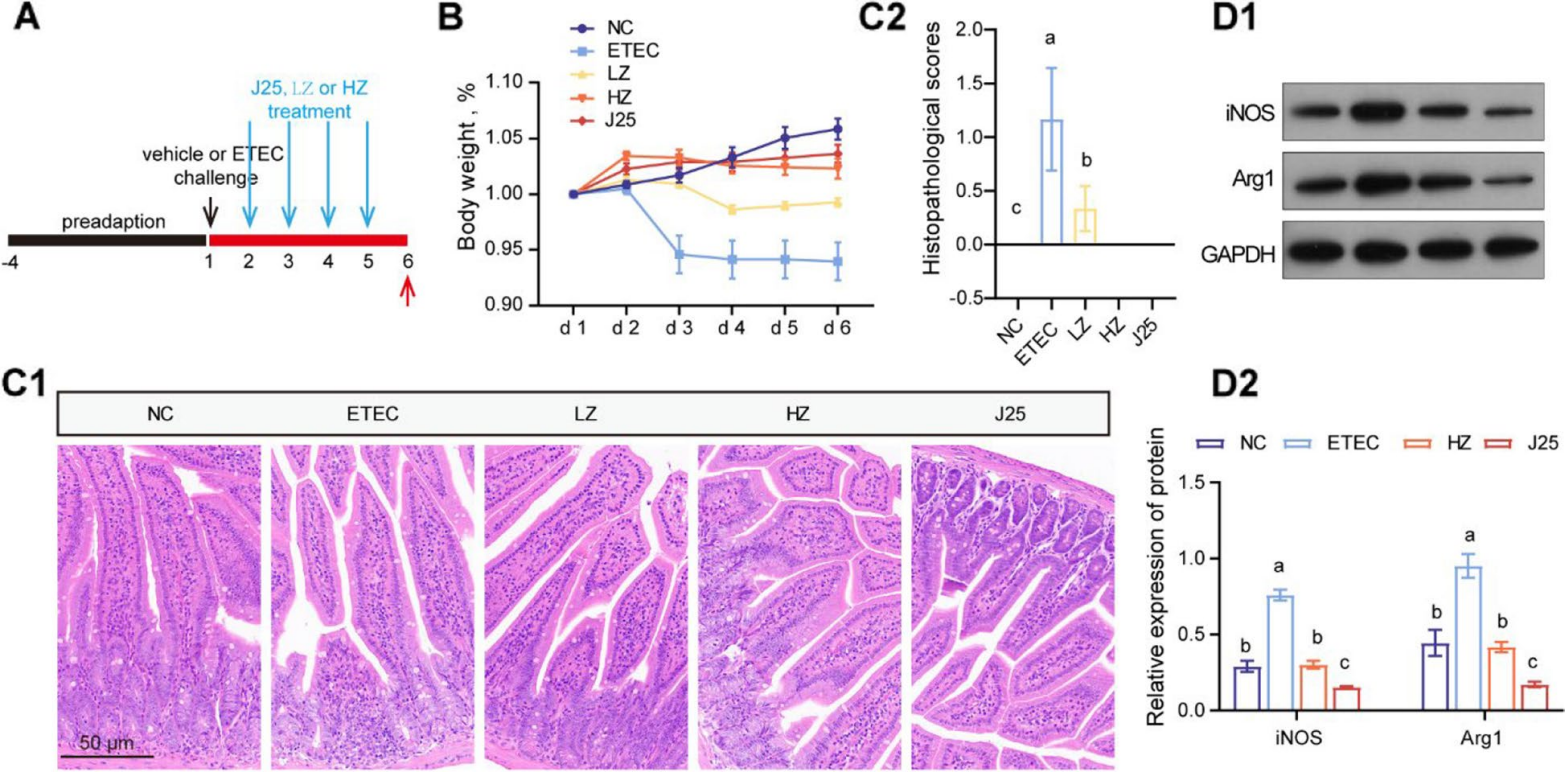
The inhibition of AA metabolism alleviated macrophage infiltration. **A** Schematic of the experimental design. Red arrows indicate the days on which the samples were collected for analysis. **B** Body weight changes (relative to original weight, set as 100%). Data are presented as mean ± SEM, *n* = 8. **C** Representative images of the jejunum by H&E staining (200×) and histopathological scores. Data are presented as mean ± SEM, *n* = 8. **D** M1 and M2 subtype macrophage relative protein levels and data statistics. Data are presented as mean ± SEM, *n* = 3. ANOVA followed by Tukey’s multiple comparisons test; different lowercase letters within each group indicate significantly different values (*P* < 0.05)

Considering that AA metabolism interacts with macrophage infiltration, we sought to determine whether this interaction relationship exists downstream of the anti-infection mechanism of J25 (Fig. 6A). The inhibitor of AA metabolism, similar to J25, alleviated weight loss and intestinal injury (Fig. 6B and C). Moreover, AA metabolism inhibition downregulated the ETEC-induced macrophage infiltration (Fig. 6D). Thus, there is interaction between AA metabolism and macrophages downstream of the anti-infective mechanism of J25 which, in turn, boosts its anti-infective effective.

## Discussion

Bacteriocins’ diversity and potency make them attractive candidates for translational application as anti-infective drugs [2, 71]. Microcin J25 can exhibit potent antibacterial activity in vitro [19, 25], but a compelling role in mammals has yet to be demonstrated. The apparent similarities between pigs and human enterotoxigenic *Escherichia coli* (ETEC) infections, and between both species, make the pig an excellent animal model. In this study, we first established a stable, symptomatically moderate ETEC challenge piglet model. Then, we demonstrated that J25 alleviated the diarrhea and intestinal injury caused by ETEC infection. Unlike gentamicin (Gen), J25 did not severely affect the gut microbial structure. This protective effect of J25 against ETEC is related to the differential regulation of dopamine receptors (DR)D1 and DRD5, and their downstream pathways. We offer the possibility that, through these pathways, J25 inhibits macrophages, enteric glial cells (EGCs), and arachidonic acid (AA) metabolism, thereby reducing inflammation. This is the first study to explain J25’s protective effect against ETEC infection in terms of immunity, the enteric nervous system (ENS), and gut microbiota.

ETEC challenge can cause impaired growth performance, diarrhea, and intestinal inflammation in piglets [21]. To verify J25’s anti-infective effect, we orally administered it to ETEC-challenged piglets. Consistent with previous studies [72–74], ETEC significantly reduced growth performance and increased both diarrhea rates and levels of TNF-α and IL-1β in serum and intestinal tissue, which were ameliorated by J25 in this study. Another feature of ETEC infection is small intestinal damage, which often results in deeper crypt depth, lower villous height and villous height/crypt depth (VCR) [41]. J25 alleviated the pathological changes in the jejunum, and this phenomenon is consistent with the significantly decreased DAO levels in serum. The morphometric evaluation showed an increase in VCR after J25 treatment. This was mainly attributable to the decrease in crypt depth, as we observed no significant differences in villous height. Moreover, the pathological improvement could also be explained by the inhibition of TNF-α and IL-1β levels.

There is growing evidence from human and animal studies that ETEC infection is associated with intestinal inflammation and innate immune activation [45–47]. The invasion of pathogenic microorganisms activates macrophages, usually leading to effective immunity [75]. Thus, it is worthwhile to investigate the responses and functions of macrophages during J25 treatment for ETEC infection. We found that the ETEC-challenged piglets’ jejunal macrophage levels were significantly upregulated, but that they were downregulated after J25 treatment. This is not surprising, since excessive or prolonged activation programs are equally harmful to the host [76]. These results were replicated in mouse models, further confirming the downregulation of macrophage function by J25. This suggests that J25, unlike proinflammatory AMPs with chemotactic functions [77, 78], plays an anti-inflammatory role in ETEC infection.

Despite the strong association between macrophages and intestinal microbiota [48, 49], we found that, unlike the gentamicin (Gen), J25 had no significant effect on microbiota structure. This is consistent with previous results, which indicate that microcin does not severely affect the microbiota [8], whereas antibiotics do [79]. It is generally believed that antibiotics can cause intestinal microbiota dysbiosis, contributing to the loss of colonization resistance and thus increasing the resistome in the intestine [80–82]. Our study does not provide direct evidence to support of any of these biological mechanisms. However, it does provide indirect evidence in support of the increased resistance after Gen treatment, based on metabolome results (Fig. S9A). In addition, the Gen-altered microbiota structure was significantly correlated with significantly altered metabolites in the vancomycin resistance pathway (Fig. S9C). These results provide preliminary in vivo evidence that bacteriocins are much more favorable than conventional antibiotics in preventing the resistance evolution.

Dopamine (DA) is a neurotransmitter that bridges the nervous and immune systems [83–85]. DA receptors (DRs) are present in most immune cells, including macrophages and dendritic cells; their different roles can be explained by their different downstream signals [86]. Our results show that J25 treatment significantly upregulated DA in ETEC-challenged piglets, and differentially regulated DRs. First, D5 and its downstream protein PKA, an inhibitor of NF-κB [55], significantly increased. NF-κB is a key signaling molecule in the activation of macrophages [58, 60, 62], and was also downregulated in our findings. Similar to our results, Wu et al. also found that DA inhibits NF-κB activation through DRD5 and different DRD5 downstream pathways, thus inhibiting inflammatory responses in macrophages [87]. Enteric glial cells (EGCs), another component of the enteric nervous system (ENS), are similar to macrophages, and are positively regulated by NF-κB and form a mutually reinforcing relationship with macrophages [59, 61, 63]. We also found that they were significantly downregulated after J25 treatment. Reactive EGCs promote inflammation in response to pathogenic microorganisms [13, 14, 18], and release proinflammatory factors such as IL-1β, which aggravate epithelial barrier dysfunction and injury [88, 89]. Therefore, NF-κB inhibition through the upregulation of D5 and its downstream PKA, and then inhibition of macrophages and EGCs, is of great significance for the regression of inflammation. In conclusion, our study provides an ENS-immune binding perspective for the anti-infective mechanism of AMPs.

Although DRD1 and DRD5 are in the same D1-class receptors, we found different trends and different anti-inflammatory mechanisms after J25 treatment. Our results suggest that D1 may inhibit downstream arachidonic acid (AA) metabolism through the DAG-PKC pathway. The downregulation of the key enzyme 5-LOX in AA metabolism further supported our hypothesis. This corroborates previous opinions suggesting that the AA metabolic network is a key target for anti-inflammatory drugs [90]. Previous studies have reported the opposite regulation of D1- and D2-class receptors [91], which was also observed in our study through the upregulation of DRD5 and the downregulation of DRD2. Moreover, we have also provided evidence of the opposite regulation among D1-class receptors. This is important because it is difficult to achieve effective resistance to inflammation with a single inflammation target.

Although bacteriocins have attracted attention as alternatives to antibiotics, to date, few studies have demonstrated their role in preventing enteric infections. Moreover, due to the use of bacteriocin-producing strains in some studies, elucidating bacteriocins’ exact contribution may be difficult. In our study, we used purified bioengineered bacteriocin Microcin J25 to overcome these limitations. Although our study is the first to show the role of the immune system, gut microbiota and ENS in mediating inflammation resolution during J25-treated ETEC infection, there are some limitations that must be considered. Our results do not provide strong evidence for the proposed pathway. Thus, additional studies are needed to verify this, especially the interaction of dopamine neurons and dopamine receptors with J25. Our preliminary data suggest that the ENS component plays an important role in preventing ETEC infection during J25 treatment, and thus, should be considered in the study of bacteriocins’ anti-infection mechanisms.

## Conclusions

**Fig. 7:**
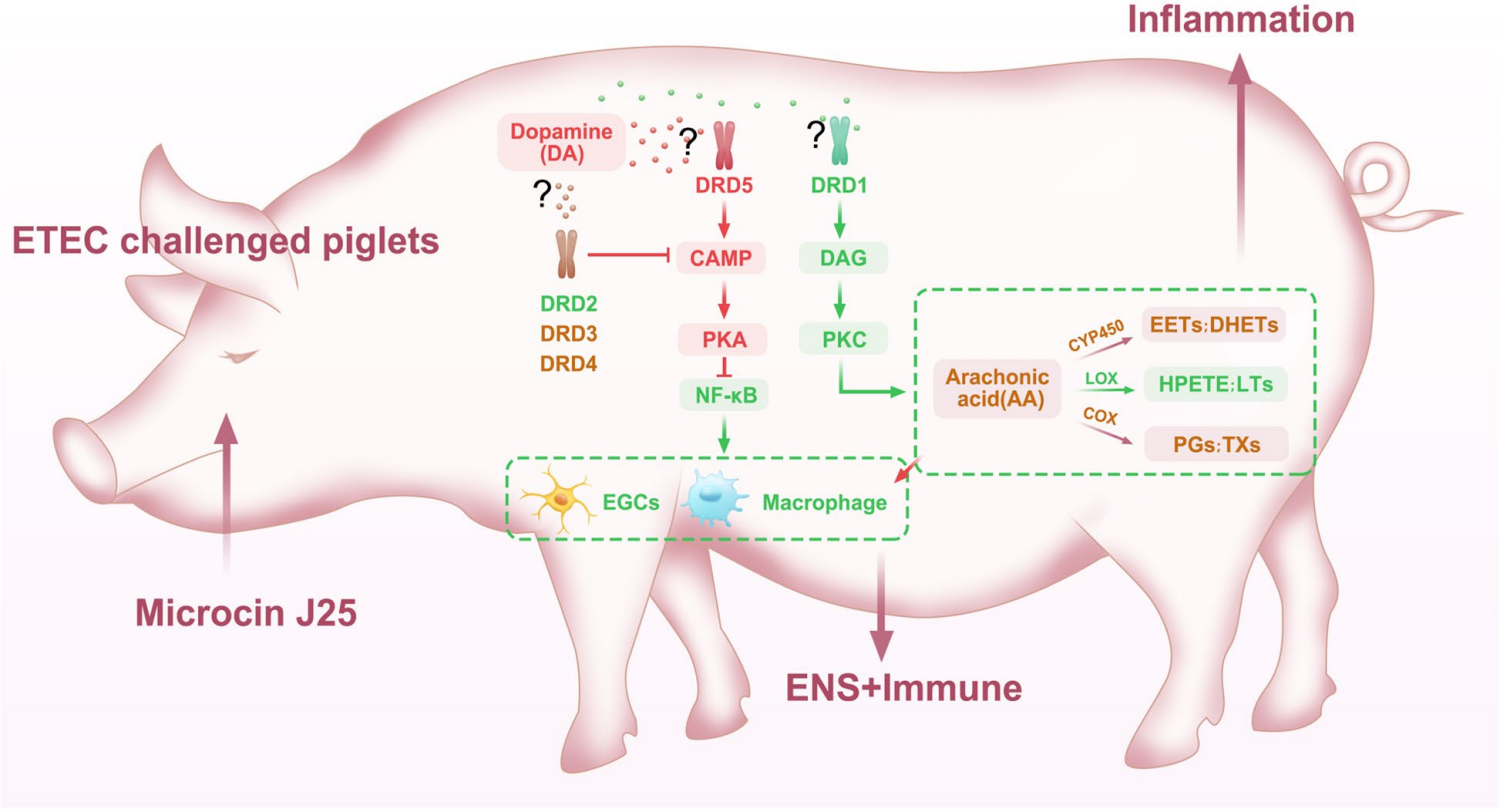
Mechanisms whereby J25 prevents ETEC infection in piglets. The administration of J25 activates cAMP-PKA-mediated NF-κB downregulation through increased DRD5 expression. This dampens EGCs and macrophages, and decrease arachidonic acid (AA) metabolism by lowering DAG-PKC via decreased DRD1, attenuating intestinal inflammation. In addition, downregulated DRD2 by J25 attenuating cAMP inhibition, whereas reinforcing the inhibitory effect of EGCs and macrophages. Reduced AA metabolism further enhanced the inhibitory effect on macrophage infiltration

In summary (Fig. 7), we demonstrated for the first time that bacteriocin Microcin J25’s anti-ETEC infection effect in piglets and a novel non-microbial regulatory mechanism that differential regulation of dopaminergic receptors. Purified bioengineered J25 was used to overcome the limitation that the use of bacteriocin-producing strains makes it difficult to elucidate bacteriocins’ exact contributions. Our preliminary data suggest that the ENS component plays an important role in preventing ETEC infection during J25 treatment, and thus, should be considered in the study of bacteriocins’ anti-infection mechanisms.

## Supplementary Information

Additional file 1: Table S1 Ingredient composition of the experimental diets. Table S2 Primers used in the study. Table S3 Scoring system for histological changes in the jejunum. Table S4 Components of the used volume densities. Table S5 Primary and secondary antibodies used for immunofluorescence and western blot labeling. Table S6 Environmental factor screening based on VIF analysis. Fig. S1 F4 susceptibility screening results. (A) For infection model establishment. (B) For J25/Gen treatment. Fig. S2 Effects of graded levels ETEC challenge on piglets. (A) Experimental design. (B) Fecal fluid content analysis. *n* = 18, mean ± SEM; *, compared with the NC group, *P* < 0.05. (C) Diarrhea score: 0, normal; 1, loose stool; 2, moderate diarrhea; 3, severe diarrhea. *n* = 18, mean ± SEM. (D) Cytokines measured in the serum on d 0, after ETEC challenge for 24 h, and on d 5. *n* = 6, mean ± SEM. (E) Total bacteria and E. coli populations in the feces. *n* = 6, mean ± SEM. ANOVA followed by Tukey’s multiple comparisons test; different lowercase letters within each group indicate significantly different values (*P* < 0.05). Fig. S3 J25’s effects on jejunum morphology, inflammation status and fecal microbiota output. (A) Morphological measurement of the jejunum. (B–C) Cytokines in the serum. (D) Cytokines in the jejunum. (E) Fecal E.coli and total bacteria detected on d 0, ETEC 24 h and d 5 by PCR. *n* = 8, mean ± SEM. ANOVA followed by Tukey’s multiple comparisons test; different lowercase letters within each group indicate significantly different values (*P* < 0.05). Fig. S4 J25’s effects on ETEC-challenged mice. (A) Experimental design of repeated trials in the mouse model. (B) Body weight changes (relative to original weight, set as 100%), *n* = 18, mean ± SEM. (C) Diarrhea score. Scoring standards: 0, normal; 1, loose stool; 2, moderate diarrhea; 3, severe diarrhea. *n* = 18, mean ± SEM. (D) Representative images of the jejunum by H&E staining and histopathological scores. *n* = 6, mean ± SEM. (E) Cytokines levels in the serum. *n* = 6, mean ± SEM. (F and G) Representative FACS plots of CD3^−^CD49b^+^ (F) and F4/80^+^ CD11b^+^ (G) staining of cells isolated from mouse jejunums, and quantification of the results. *n* = 4, mean ± SEM. ANOVA followed by Tukey’s multiple comparisons test; different lowercase letters within each group indicate significantly different values (*P *< 0.05). Fig. S5 Relative abundance of predicted metabolic potential of microbes from ETEC and ETEC + J25 groups, as predicted by PICRUSt2. Fig. S6 Correlation matrix between microbiota and important parameters. Only genera with statistically significantly change are shown. Spearman correlation, *, *P* < 0.05; **, *P *< 0.01; ***, *P *< 0.001. Fig. S7 Significantly altered metabolites in neuron-related pathways identified by the metabolome. (A–F) Metabolites in the dopaminergic neuron pathway. (C, E–H) Metabolites in the serotonergic neuron pathway. (C, D, I, J) Metabolites in the glutamatergic neuron pathway. (I, J) Metabolites in the GABAergic neuron pathway. (C, K, L) Metabolites in the cholinergic neuron pathway. *n* = 8, mean ± SEM. ANOVA followed by Dunnett’s multiple comparisons test; different lowercase letters within each group indicate significantly different values (*P *< 0.05). Fig. S8 Effects of graded levels of 6-OHDA lesions on mice. (A) Body weight changes (relative to original weight, set as 100%), d 1, *n* = 12; d 2–7, *n* = 6, mean ± SEM. (B) Representative images of TH immunoreactivity by the immunohistochemistry method in the jejunum on d 1 (first, top of B1) and d 6 (second, bottom of B1), and relative immunolabeling quantification (B2). (C) Representative images of the jejunum by H&E staining on d 6 and histopathological scores. *n* = 6, mean ± SEM. ANOVA followed by Tukey’s multiple comparisons test; different lowercase letters within each group indicate significantly different values (*P* < 0.05). Fig. S9 Gentamicin-induced resistance was significantly associated with altered microbiota structure. (A) Annotation results of differential metabolites between ETEC and Gen groups. (B) Differential metabolites in the vancomycin resistance pathway. *n* = 8, mean ± SEM. ANOVA followed by Dunnett’s multiple comparisons test; different lowercase letters within each group indicate significantly different values (*P *< 0.05). (C) Canonical Correspondence Analysis plot. The arrow length represents the strength of the correlation between the environmental variables and the microbes. The longer the arrow, the stronger the correlation. The perpendicular distance between the microbes and the environmental variable axes on the plot reflects their correlations. The less the distance, the stronger the correlation.

## Abbreviations

AMPs: Antimicrobial peptides
DR: Dopamine receptor
ENS: Enteric nervous system
ETEC: Enterotoxigenic *Escherichia coli*
Gen: Gentamicin
J25: Microcin J25
LB: Luria-bertani
OTUs: Operational taxonomic units
TPH: Tryptophan hydroxylase
6-OHDA: 6-Hydroxydopamine
