# Supplementary material for: Bacteriocin Microcin J25’s antibacterial infection effects and novel non-microbial regulatory mechanisms: differential regulation of dopaminergic receptors
Source: J Anim Sci Biotechnol. 2024 Nov 13;15:156. doi: 10.1186/s40104-024-01115-3 (PMC11559059; doi:10.1186/s40104-024-01115-3)
Supplement: Supplementary file 1 — Additional file 1: Table S1 Ingredient composition of the experimental diets. Table S2 Primers used in the study. Table S3 Scoring system for histological changes in the jejunum. Table S4 Components of the used volume densities. Table S5 Primary and secondary antibodies used for immunofluorescence and western blot labeling. Table S6 Environmental factor screening based on VIF analysis. Fig. S1 F4 susceptibility screening results. (A) For infection model establishment. (B) For J25/Gen treatment. Fig. S2 Effects of graded levels ETEC challenge on piglets. (A) Experimental design. (B) Fecal fluid content analysis. n = 18, mean ± SEM; *, compared with the NC group, P < 0.05. (C) Diarrhea score: 0, normal; 1, loose stool; 2, moderate diarrhea; 3, severe diarrhea. n = 18, mean ± SEM. (D) Cytokines measured in the serum on d 0, after ETEC challenge for 24 h, and on d 5. n = 6, mean ± SEM. (E) Total bacteria and E. coli populations in the feces. n = 6, mean ± SEM. ANOVA followed by Tukey’s multiple comparisons test; different lowercase letters within each group indicate significantly different values (P < 0.05). Fig. S3 J25’s effects on jejunum morphology, inflammation status and fecal microbiota output. (A) Morphological measurement of the jejunum. (B–C) Cytokines in the serum. (D) Cytokines in the jejunum. (E) Fecal E.coli and total bacteria detected on d 0, ETEC 24 h and d 5 by PCR. n = 8, mean ± SEM. ANOVA followed by Tukey’s multiple comparisons test; different lowercase letters within each group indicate significantly different values (P < 0.05). Fig. S4 J25’s effects on ETEC-challenged mice. (A) Experimental design of repeated trials in the mouse model. (B) Body weight changes (relative to original weight, set as 100%), n = 18, mean ± SEM. (C) Diarrhea score. Scoring standards: 0, normal; 1, loose stool; 2, moderate diarrhea; 3, severe diarrhea. n = 18, mean ± SEM. (D) Representative images of the jejunum by H&E staining and histopathological scores. n = 6, mean ± SE [file 40104_2024_1115_MOESM1_ESM.docx]

**Table S1** Ingredient composition of the experimental diets (%, as­fed basis)

| **Ingredient** | **Diet** |
| --- | --- |
| Corn | 52.42 |
| Extruded corn | 10.00 |
| Soybean meal | 13.11 |
| Extruded full-fat soybean | 11.00 |
| Whey powder | 5.00 |
| Fish meal | 4.00 |
| Soybean oil | 1.60 |
| Dicalcium phosphate | 0.63 |
| Limestone | 0.59 |
| Salt | 0.30 |
| Permix ^1^ | 0.50 |
| Lys | 0.36 |
| Met | 0.14 |
| Thr | 0.10 |
| Trp | 0.05 |
| Choline chloride | 0.20 |
| Calculated composition |  |
| Net energy, kcal/kg | 2,618 |
| Crude protein | 17.71 |
| Ca | 0.65 |
| P | 0.57 |
| SID Lys, % | 1.35 |
| SID Met+Cys, % | 0.78 |
| SID Thr, % | 0.80 |
| SID Trp, % | 0.25 |

**Table S2** Primers used in the study

| **Primer** | **Nucleotide sequence (5′-3′)** |
| --- | --- |
| MUC4 |  |
| Forward | GTGCCTTGGGTGAGAGGTTA |
| Reverse | CACTCTGCCGTTCTCTTTCC |
| Total bacteria |  |
| Forward | ACTCCTACGGGAGGCAGCAG |
| Reverse | ATTACCGCGGCTGCTGG |
| *Escherichia coli* |  |
| Forward | CATGCCGCGTGTATGAAGAA |
| Reverse | CGGGTAACGTCAATGAGCAAA |
| Probe | AGGTATTAACTTTACTCCCTTCCTC |
| DRD1 |  |
| Forward | CACAAGGCAAAACCCACGAG |
| Reverse | TCTGGTTGAGAACACTCCGC |
| DRD2 |  |
| Forward | CCAGACCAGAGAAGAACGGAC |
| Reverse | GTTTTGCCATTGGGCATGGA |
| DRD3 |  |
| Forward | ATGCCATGGGGAAAACGGAAC |
| Reverse | GCTGCAGGTGTAACAAGAGAGA |
| DRD4 |  |
| Forward | CATCAGCGTGGACAGGTTCGT |
| Reverse | AAGGAGCACACGGACGAGTA |
| DRD5 |  |
| Forward | CACAGGGACAAGGTAGGCTC |
| Reverse | GGAGATGGCGTAAGTTCGGT |
| β-actin |  |
| Forward | AATCCTGCGGCATCCACGAAAC |
| Reverse | CAGCACCGTGTTGGCGTAGAG |

**Table S3** Scoring system for histological changes in the jejunum

| **Score** | **Inflammatory** | **Epithelium** | **Lesion** | **Lesion degree** |
| --- | --- | --- | --- | --- |
| 0 | None | None | None | None |
| 1 | Lamina propria inflammatory mild infiltration | Isolated focal epithelial damage | Mucosal layer | 10-25% |
| 2 | Mild infiltration and multifocal edema | Local injury and epithelial cytopenia | Mucosa and submucosa | 26-50% |
| 3 | Extensive inflammatory infiltration, glandular separation and edema | Extensive damage deep into the bowel wall | Transmural | ＞50% |

**Table S4** Components of the used volume densities

| **Vv** | **Space of interest** | **Reference space** |
| --- | --- | --- |
| GFAP in muscularis | GFAP IR in muscularis | β3-Tubulin IR+ GFAP IR in muscularis |
| GFAP in submucosa | GFAP IR in submucosa | β3-Tubulin IR+ GFAP IR in submucosa |

IR, immunoreactivity by immunofluorescence

**Table S5** Primary and secondary antibodies used for immunofluorescence and western blot labeling

| **Antigen** | **Host** | **Dilution** | **Source** | **Cat#/RRID** |
| --- | --- | --- | --- | --- |
| F4/80 | Rabbit | 1:500 | Proteintech | 28463-1-AP |
| CD86 | Rabbit | 1:100 | Abcam | ab269587 |
| Mannose | Rabbit | 1:100 | Genetex | GTX53806 |
| CD3 | Rabbit | 1:200 | Proteintech | 17617-1-AP |
| CD49b | Mouse | 1:100 | MAB | MAB1998 |
| Tubulin | Mouse | 1:100 | Proteintech | CL594-66240 |
| GFAP | Rabbit | 1:100 | Proteintech | 23935-1-AP |
| IgG | Goat | 1:400 | SeraCare | 5220-0362 |
| IgG | Rabbit | 1:400 | SeraCare | 5220-0336 |
| IgG | Mouse | 1:400 | SeraCare | 5220-0341 |
| IgG | Rat | 1:200 | SeraCare | 5220-0364 |
| GAPDH | Rabbit | 1:10,000 | Proteintech | 10494-1-AP |
| ALDH1A1 | Mouse | 1:500 | Proteintech | 60171-1-lg |
| GAT1 | Rabbit | 1:1,000 | abcam | ab177483 |
| PKA | Rabbit | 1:1,000 | Cell Signaling | 4781 |
| PKC | Rabbit | 1:2,000 | Proteintech | 21991-1-AP |
| p65 | Rabbit | 1:1,000 | Cell Signaling | 3033 |
| p-p65 | Mouse | 1:1,000 | Cell Signaling | 6956 |
| Cytp450 | Rabbit | 1:500 | Bioss | Bs-14150R |
| 5-Lox | Mouse | 1:4,000 | Proteintech | 66326-1-lg |
| Cox | Rabbit | 1:2,000 | absin | Abs131985 |
| IgG | Mouse | 1:10,000 | Proteintech | SA00001-1 |
| IgG | Rabbit | 1:10,000 | Proteintech | SA00001-2 |

**Table S6** Environmental factor screening based on VIF analysis

| **Environmental factor** | **VIF** |
| --- | --- |
| Fecal fluid content | 1.42 |
| Macrophages | 6.13 |
| M1 subtypes | 4.20 |
| M2 subtypes | 3.02 |


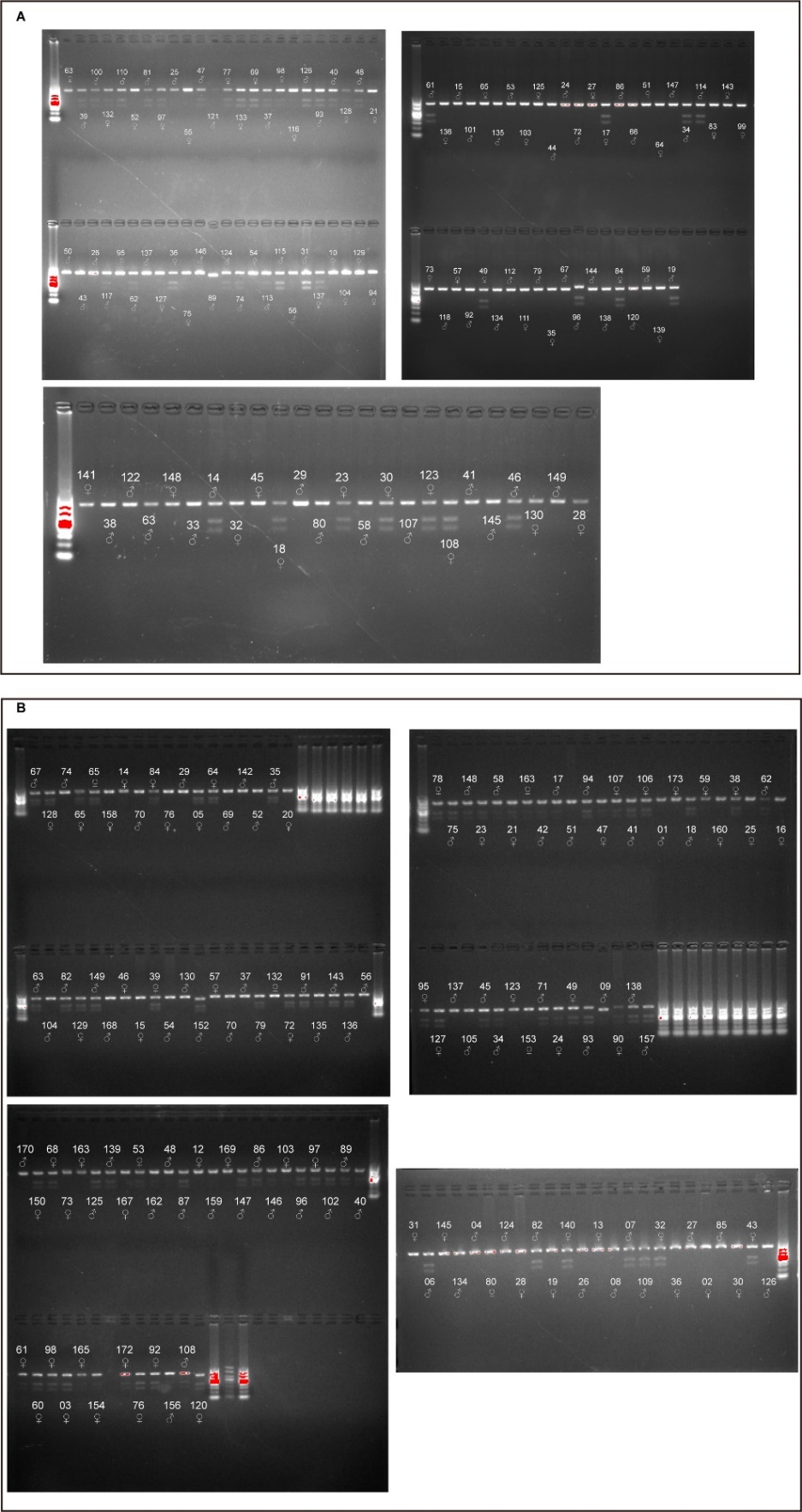
**Fig. 1** F4 susceptibility screening results. **A** For infection model establishment. **B** For J25/Gen treatment


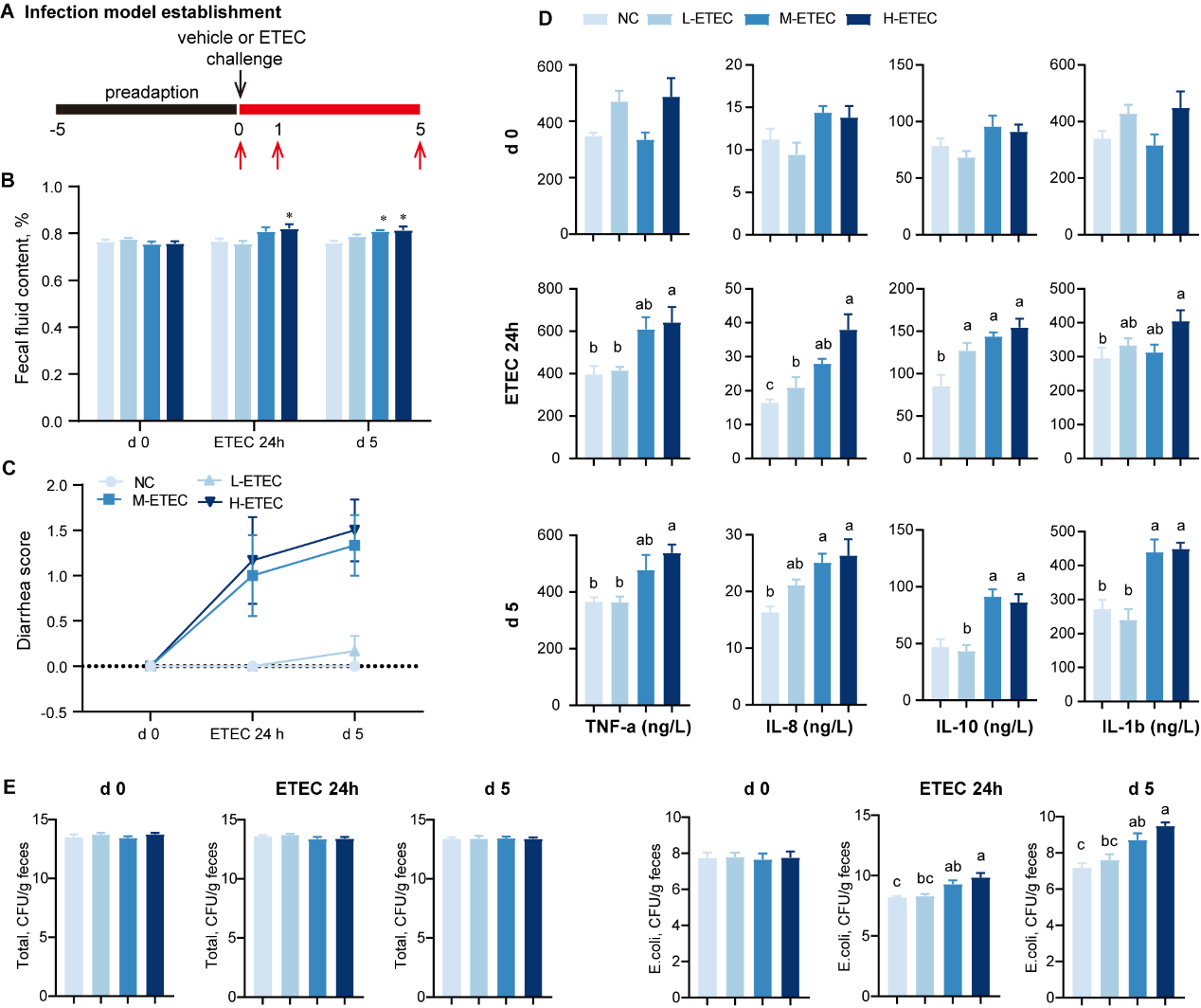
**Fig. 2** Effects of graded levels ETEC challenge on piglets. **A** Experimental design. **B** Fecal fluid content analysis. Data are presented as mean ± SEM, *n* = 18; *, compared with the NC group, *P*＜0.05. **C** Diarrhea score: 0, normal; 1, loose stool; 2, moderate diarrhea; 3, severe diarrhea. Data are presented as mean ± SEM, *n* = 18. **D** Cytokines measured in the serum on d 0, after ETEC challenge for 24 h, and on d 5. Data are presented as mean ± SEM, *n* = 6. **E** Total bacteria and *E. coli* populations in the feces. Data are presented as mean ± SEM, *n* = 6. ANOVA followed by Tukey’s multiple comparisons test; different lowercase letters within each group indicate significantly different values (*P*＜0.05)


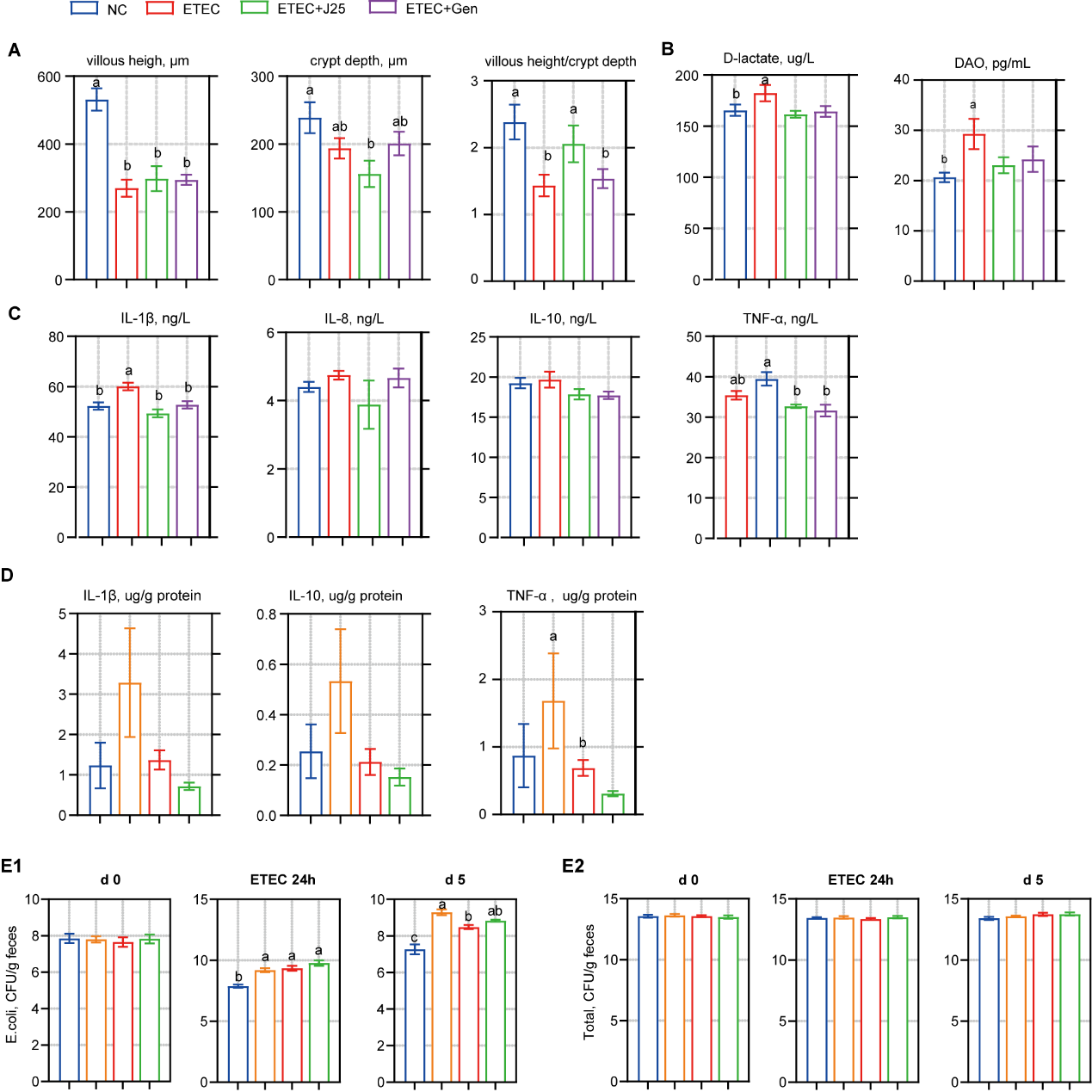
**Fig. 3** J25’s effects on jejunum morphology, inflammation status and fecal microbiota output. **A** Morphological measurement of the jejunum. **B and C** Cytokines in the serum. **D** Cytokines in the jejunum. **E** Fecal *E.coli* and total bacteria detected on d 0, ETEC 24 h and d 5 by PCR. Data are presented as mean ± SEM, *n* = 8. ANOVA followed by Tukey’s multiple comparisons test; different lowercase letters within each group indicate significantly different values (*P*＜0.05)


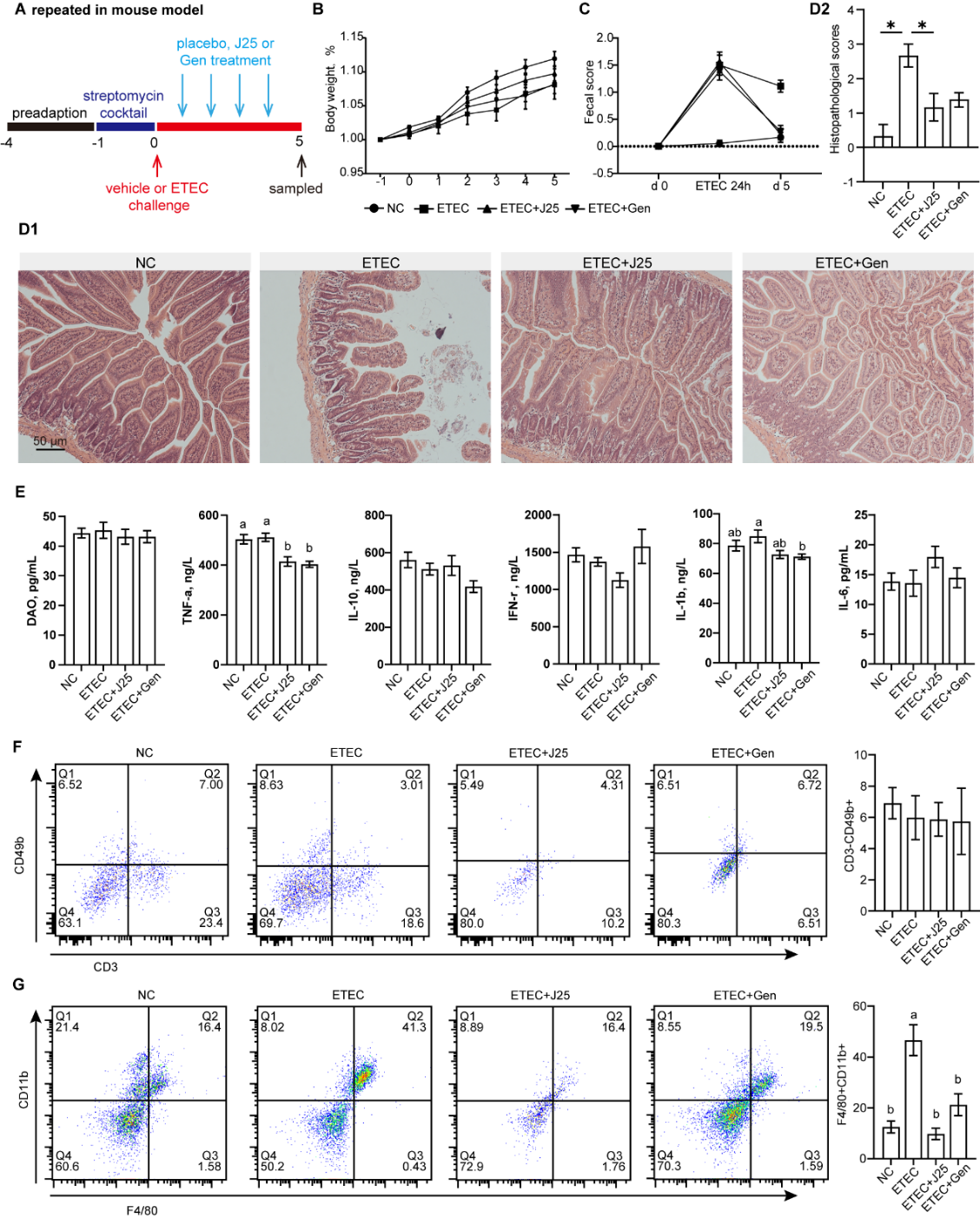
**Fig. 4** J25’s effects on ETEC-challenged mice. **A** Experimental design of repeated trials in the mouse model. **B** Body weight changes (relative to original weight, set as 100%). Data are presented as mean ± SEM, *n* = 18. **C** Diarrhea score. Scoring standards: 0, normal; 1, loose stool; 2, moderate diarrhea; 3, severe diarrhea. Data are presented as mean ± SEM, *n* = 18. **D** Representative images of the jejunum by H&E staining and histopathological scores. Data are presented as mean ± SEM, *n* = 6. **E** Cytokines levels in the serum. Data are presented as mean ± SEM, *n* = 6. **F and G** Representative FACS plots of CD3^−^CD49b^+^ (**F**) and F4/80^+^CD11b^+^ (**G**) staining of cells isolated from mouse jejunums, and quantification of the results. Data are presented as mean ± SEM, *n* = 4. ANOVA followed by Tukey’s multiple comparisons test; different lowercase letters within each group indicate significantly different values (*P*＜0.05)


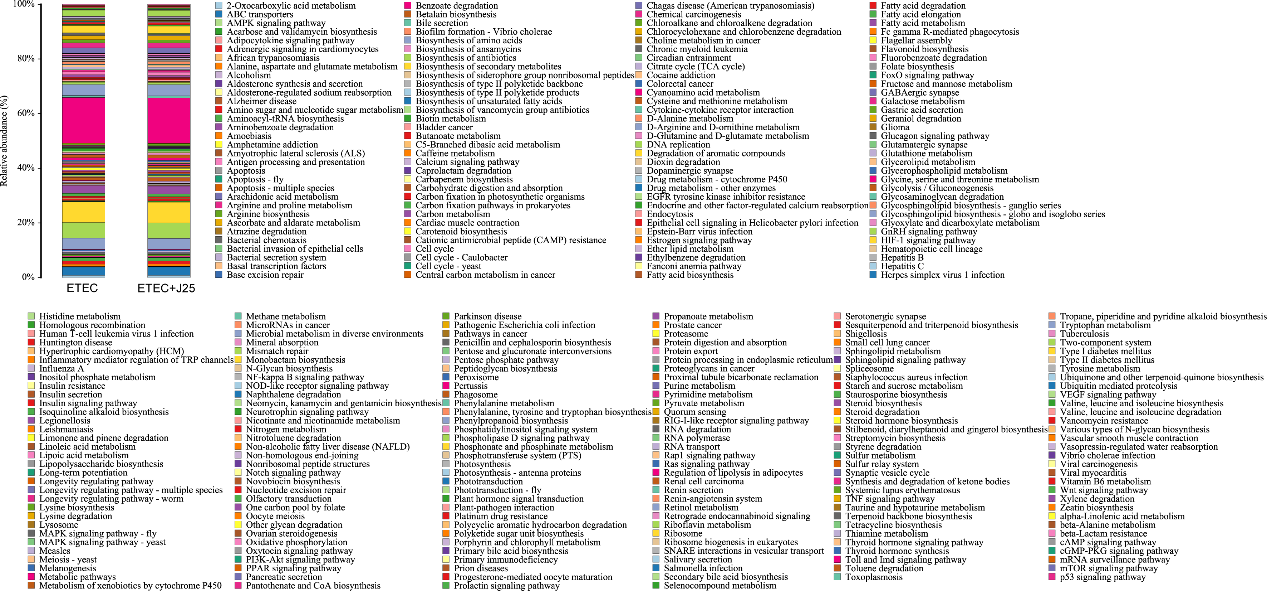
**Fig. 5** Relative abundance of predicted metabolic potential of microbes from ETEC and ETEC+J25 groups, as predicted by PICRUSt2


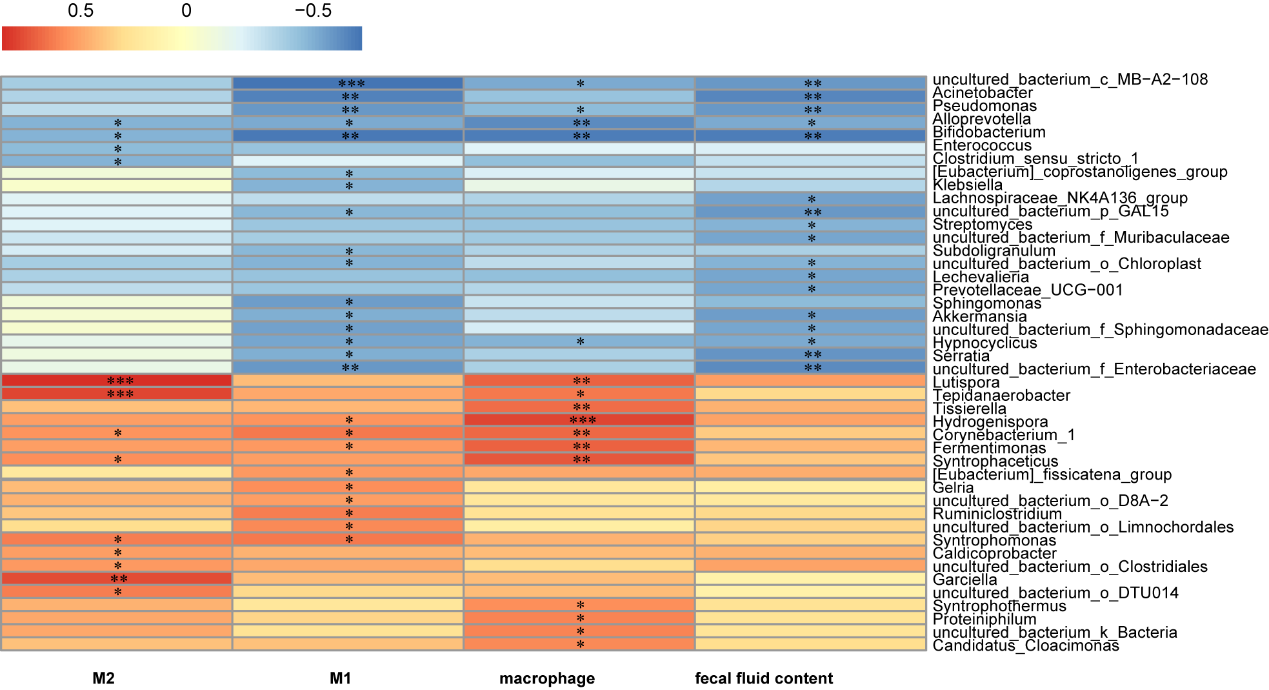


**Fig. 6** Correlation matrix between microbiota and important parameters. Only genera with statistically significantly change are shown. Spearman correlation, *, *P*＜0.05; **, *P*＜0.01; ***, *P*＜0.001


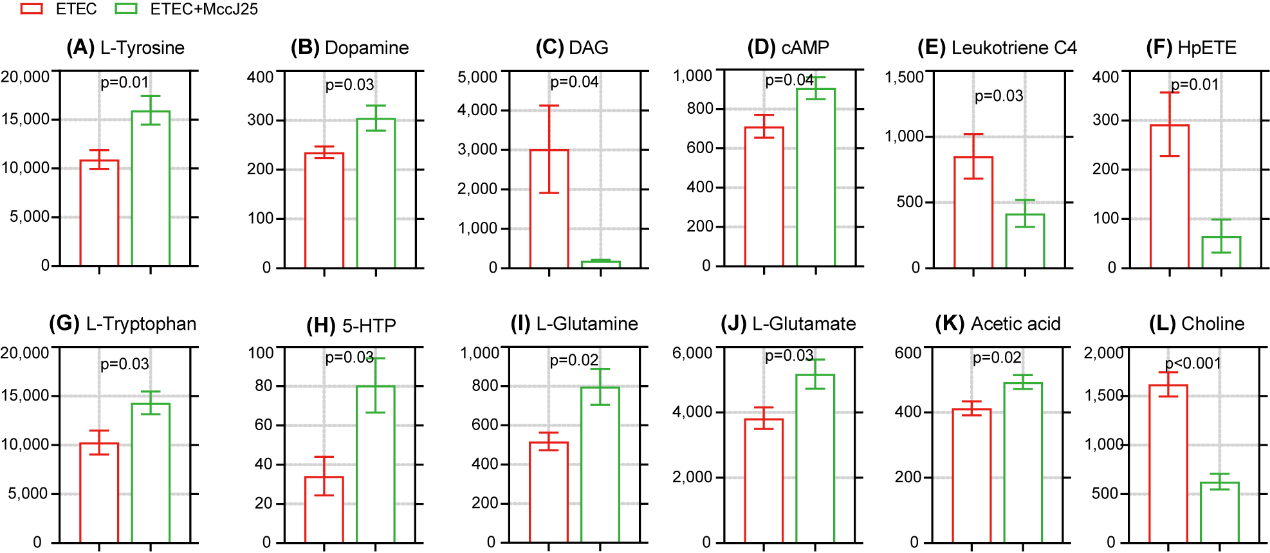
**Fig. 7** Significantly altered metabolites in neuron-related pathways identified by the metabolome. **A–F** Metabolites in the dopaminergic neuron pathway. (**C, E–H**) Metabolites in the serotonergic neuron pathway. (**C, D, I, J**) Metabolites in the glutamatergic neuron pathway. **I and J** Metabolites in the GABAergic neuron pathway. (**C, K, L**) Metabolites in the cholinergic neuron pathway. Data are presented as mean ± SEM, *n* = 8. ANOVA followed by Dunnett’s multiple comparisons test; different lowercase letters within each group indicate significantly different values (*P*＜0.05)


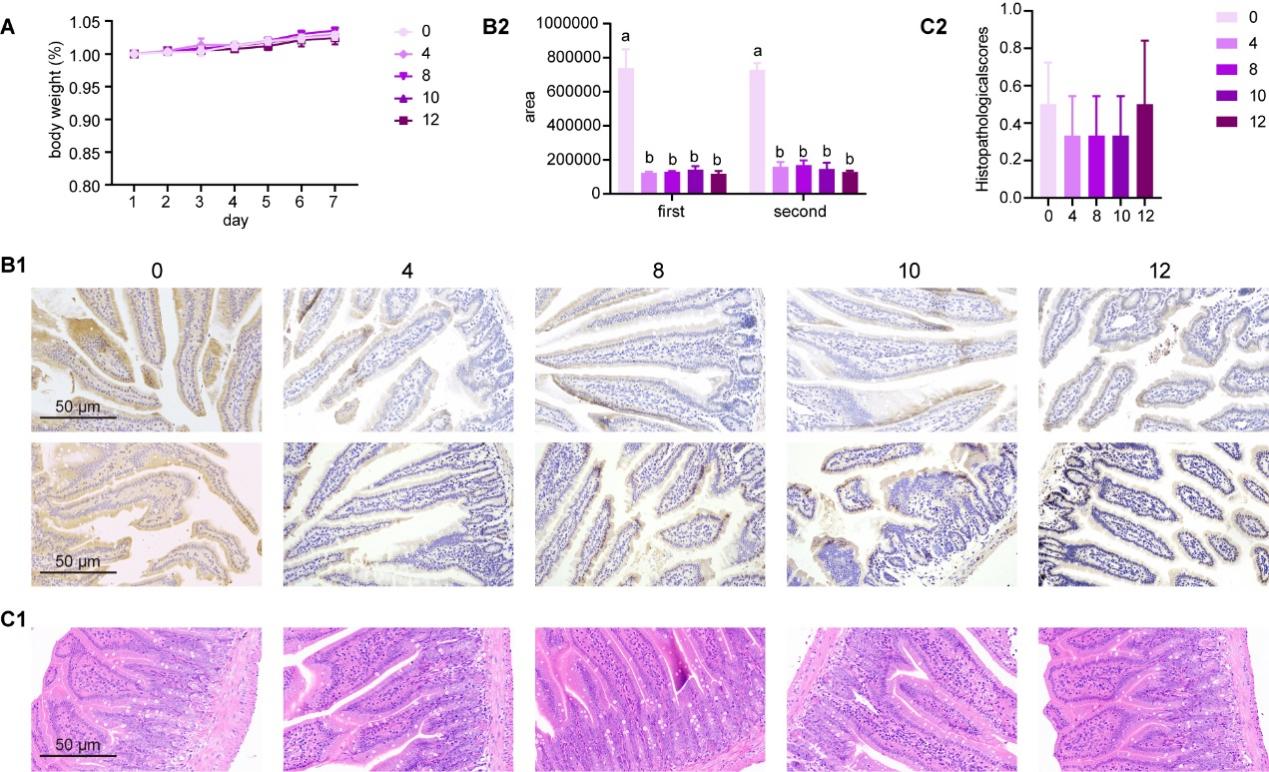
**Fig. 8** Effects of graded levels of 6-OHDA lesions on mice. **A** Body weight changes (relative to original weight, set as 100%), d 1, *n* = 12; d 2–7, *n* = 6, data are presented as mean ± SEM. **B** Representative images of TH immunoreactivity by the immunohistochemistry method in the jejunum on d 1 (first, top of B1) and d 6 (second, bottom of B1), and relative immunolabeling quantification (B2). **C** Representative images of the jejunum by H&E staining on d 6 and histopathological scores. Data are presented as mean ± SEM, *n* = 6. ANOVA followed by Tukey’s multiple comparisons test; different lowercase letters within each group indicate significantly different values (*P*＜0.05)

**
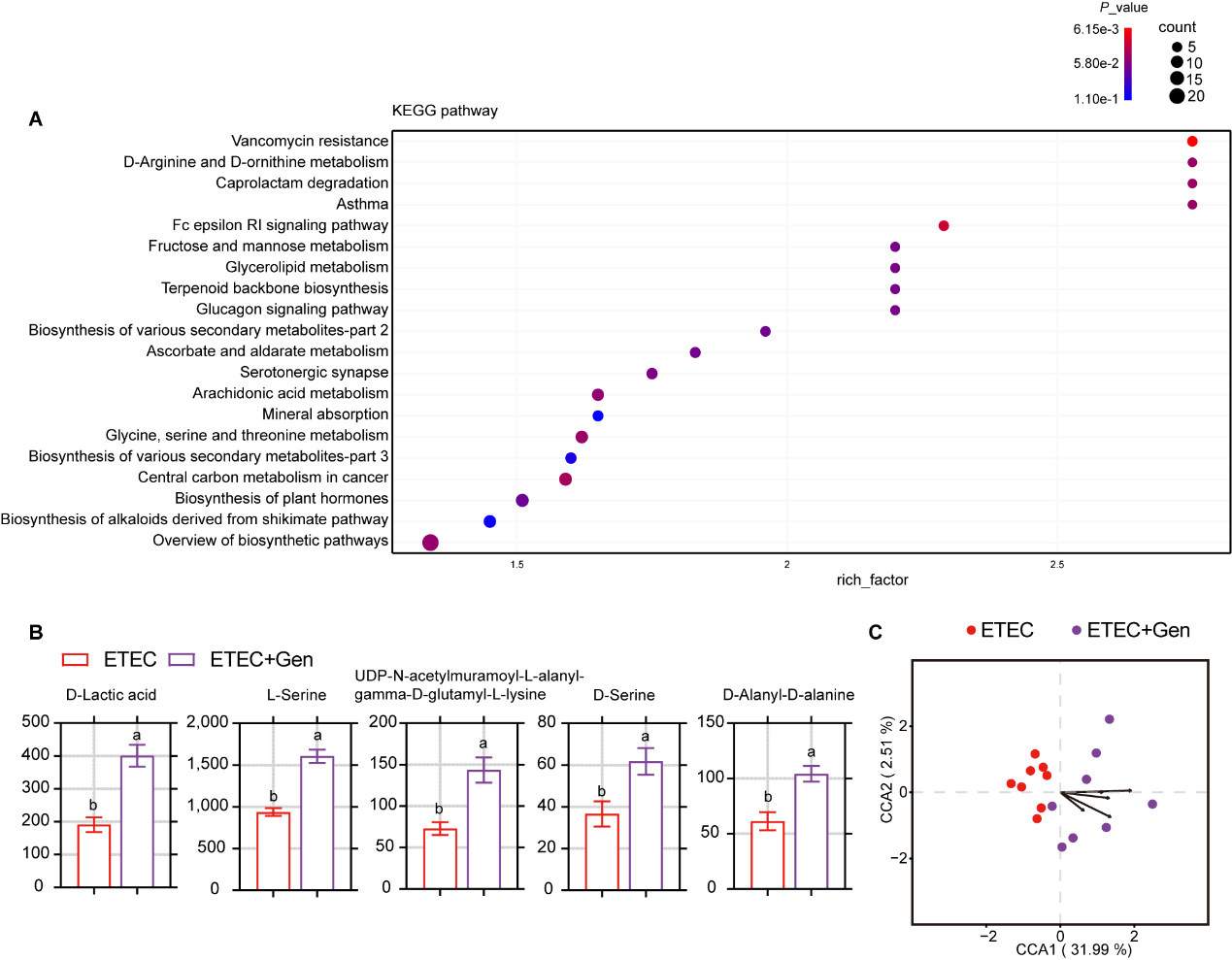
Fig. 9** Gentamicin-induced resistance was significantly associated with altered microbiota structure. **A** Annotation results of differential metabolites between ETEC and Gen groups. **B** Differential metabolites in the vancomycin resistance pathway. Data are presented as mean ± SEM, *n* = 8. ANOVA followed by Dunnett’s multiple comparisons test; different lowercase letters within each group indicate significantly different values (*P* < 0.05). **C** Canonical Correspondence Analysis plot. The arrow length represents the strength of the correlation between the environmental variables and the microbes. The longer the arrow, the stronger the correlation. The perpendicular distance between the microbes and the environmental variable axes on the plot reflects their correlations. The less the distance, the stronger the correlation
